# Mutual Destruction of Deep Lung Tumor Tissues by Nanodrug‐Conjugated Stealth Mesenchymal Stem Cells

**DOI:** 10.1002/advs.201700860

**Published:** 2018-02-26

**Authors:** Sang‐Woo Kim, Yeon Kyung Lee, Jeong Hee Hong, Jun‐Young Park, Young‐Ae Choi, Dong Un Lee, Jungil Choi, Sun Jin Sym, Sang‐Hyun Kim, Dongwoo Khang

**Affiliations:** ^1^ Lee Gil Ya Cancer and Diabetes Institute Gachon University Incheon 21999 South Korea; ^2^ Department of Physiology School of Medicine Gachon University Incheon 21999 South Korea; ^3^ Division of Hematology and Oncology School of Medicine Gachon University and Gil Hospital Incheon 21565 South Korea; ^4^ Gyeongnam Department of Environmental Toxicology and Chemistry Korea Institute of Toxicology Jinju 52834 South Korea; ^5^ Department of Pharmacology School of Medicine Kyungpook National University Daegu 41566 South Korea

**Keywords:** homing abilities, in vivo safety, lung tumors, mesenchymal stem cells, mutual destruction, nanodrug membrane conjugation

## Abstract

Lung cancer is a highly malignant tumor, and targeted delivery of anti‐cancer drugs to deep lung tumor tissue remains a challenge in drug design. Here, it is demonstrated that bone marrow mesenchymal stem cells armed with nanodrugs are highly targeted and mutually destructive with malignant lung cancer cells and successfully eradicate lung tumors tissues. Using this approach, the current clinical dose of anti‐cancer drugs for the treatment of malignant lung tumors can be decreased by more than 100‐fold without triggering immunotoxicity.

## 1 Introduction

The 10‐year survival rate of cancer patients has greatly increased over the last 20 years.^1^ However, the survival rate of patients with major malignant tumors, such as lung and pancreatic cancers, still shows negligible improvement.^1^ The conventional chemotherapeutic strategy for treating lung cancer remains unsatisfactory in this respect. Specifically, the 5‐year survival rates of both non‐small‐cell lung cancer (NSCLC) and small cell lung cancer (SCLC) patients exhibited merely 4–5% improvements in the past decade for cancer in stages I–III.^2^ The main difficulty in treating lung cancer is the lack of targeted chemotherapeutic agents specific to the lung tumor tissues and, thus, non‐specific targeting generally leads to unwanted side effects induced by excessive doses of chemotherapeutic agents.^3^

Several strategies have been introduced to resolve the aforementioned issues, such as cancer antigen‐targeted monoclonal antibody (mAb) therapy,^4^ nanodrug delivery,^5^ and cell‐mediated therapy (i.e., T‐cell‐based therapy for NSCLC^6^). However, many clinical trials have shown that intravenously administered mAb, particularly epidermal growth factor receptor‐targeting mAbs, induced dermatological toxicities (e.g., acneiform eruption, xerosis, telangiectasia, hyperpigmentation, fissures, and hair/nail changes) in over 50% of patients after treatment.^7^

Nanodrug delivery is an attractive alternative strategy for targeting lung tumors. For example, liposomal and polymeric nanoparticles containing chemotherapeutic drugs (e.g., Lipoplatin, Doxil, Abraxane, BIND‐014, Genexol‐PM, and Nanoplatin) have been used for the treatment of lung cancer.^5^ However, one drawback of inorganic‐based nanodrugs (both FDA‐approved and those in clinical trials) is the accumulation in the reticuloendothelial system and renal clearance issues.^8^

Cell‐mediated therapy offers many advantages compared to conventional nanocarriers, such as evasion of the immune system, homing to the tumor, and crossing impermeable endothelial barriers.^9^ For the treatment of NSCLC, a therapeutic approach based on genetically engineered T‐cells has been well studied and some T‐cell therapies are currently in phases I–II of clinical trials.^10^ In addition to T cells, mesenchymal stem cells (MSCs) may serve as another promising cell therapy vector due to their intrinsic homing nature to tumors.^11^ MSCs comprise several lineages, such as bone marrow MSCs (BM‐MSCs), adipose MSCs, and umbilical cord blood MSCs. The intrinsic homing ability of MSCs to cancer mainly originates from the activation of tumor‐associated chemokine receptors on MSCs.^12^, ^13^ While the influence of MSCs in determining the fate of tumors, in addition to their targeting abilities, is currently debated, it is certain that MSCs possess a homing ability toward specific tumors and their targeting ability can be optimized by selecting the appropriate type(s) of MSCs for targeting a given type of carcinoma.^11^, ^14^ Previous studies have shown successful tumor treatment using genetically engineered MSCs.^15^ Despite this success, the debated safety of genetically engineered MSCs have hindered FDA endorsement.^15^, ^16^, ^17^

To circumvent the need for genetic engineering, intracellular drug‐loaded MSCs have been the most conventional strategy for increasing the anti‐cancer efficacy of MSCs.^18^, ^19^ However, in spite of its facile approach, there are several critical drawbacks. First, the intracellularly loaded drug diminished the MSC functionality (i.e., their homing ability to cancer cells) and altered the fate of the MSCs.^16^ Second, the amount of drug that can be intracellularly loaded in MSCs is limited.^16^

In addition, drug conjugation to MSC membranes is an another advantageous strategy because it will not only minimize the uptake of drugs in MSCs,^19^ but also coat a greater amount of the drug onto the surface of MSCs through membrane‐receptor conjugation. Unfortunately, despite the therapeutic prospects of this strategy, MSC membrane conjugation has not been fully optimized for cellular function. There remains no comprehensive understanding of fate of MSC after membrane conjugation and the functional stability of MSCs after conjugation. Furthermore, no direct comparative studies have been performed for treating tumors, including studies between intracellularly loaded MSCs and membrane‐conjugated MSCs. Perhaps most importantly, there has been no successful report on any organ‐specific tumor model (i.e., deep‐tissue tumor model) aside from the skin xenograft model that uses MSC membrane‐conjugated anti‐cancer drugs.^19^

In light of these reasons, the present study introduces a systematic strategy for the elimination of lung tumors (i.e., lung tumor‐bearing mice) using lung‐tumor‐targeted nanodrugs conjugated on MSCs. In addition, for the first time, this study provides mutual destruction mechanism of lung tumor cells by nanodrug conjugated mesenchymal stem cells.

## 2 Results and Discussion

### 2.1 Bone Marrow MSCs Robustly Target Lung Cancer Cells

**Figure 1:**
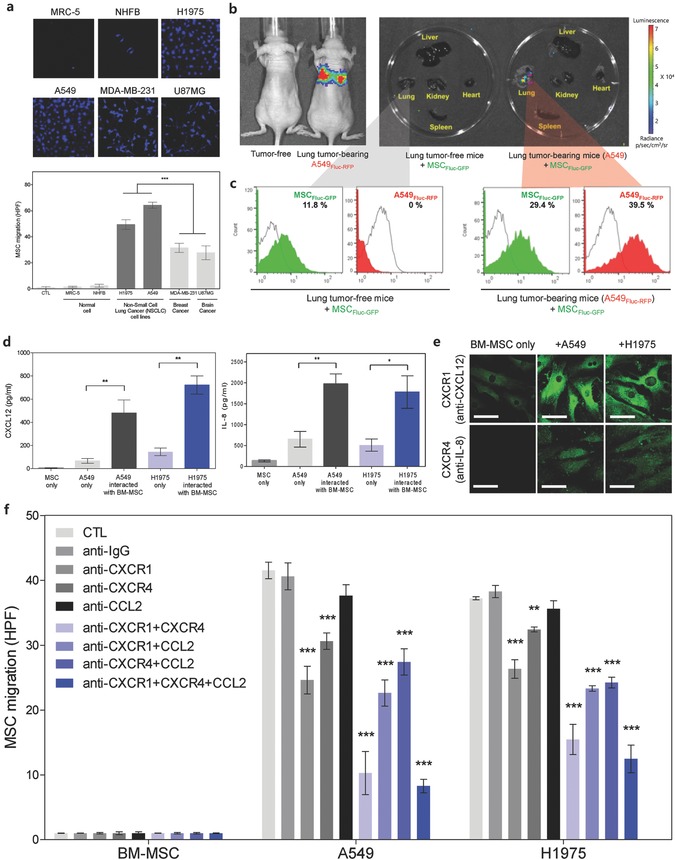
BM‐MSCs homing to lung cancer, and determination of homing‐related cytokine receptors on MSCs. a) Differential in vitro tropism toward various cancer cells based on MSC lineages. Inset images are the representative confocal images of the transwell migration of BM‐MSC toward normal and cancer cells (i.e., lung, breast, and brain cancer). BM‐MSCs showed accelerated homing ability toward human lung cancer cells (e.g., H1975 and A549). Data represent mean ± standard error of the means (SEM) (*n* = 10). b) Bioluminescence image (left) of tumor‐free mice and lung tumor‐bearing (A549_Fluc–RFP_) mice. Ex vivo imaging (right) of major organs (liver, lung, kidney, heart, and spleen) harvested from tumor‐free and lung tumor‐bearing (A549) mice at 3 d after the intravenous injection of MSC_Fluc–GFP_. Tissues were imaged within 3 min of explanting. c) FACS histogram analysis shows increased percentage of MSC_Fluc–GFP_ in A549_Fluc–RFP_ tumor bearing mice compared to tumor free mice (in the lung tissue) after 2 d of the intravenous injection of MSC_Fluc–GFP_. d) Detection of chemokines (CXCL12 and IL‐8) secreted from A549 and H1975 lung cancer cells when co‐cultured with BM‐MSCs. CXCL12 and IL‐8 chemokine levels quantitated by ELISA. Data represent mean ± SEM (*n* = 3), *p < 0.05 and **p < 0.01 versus A549 cells (48 h). e) Confocal images represent phenotypic expression of MSC cytokine receptors (CXCR1 and CXCR4) in response to CXCL12 and IL‐8 secreted from A549 and H1975. f) BM‐MSC migration toward lung cancer cells (A549 and H1975) after blocking chemokine receptors on the MSC surface. MSCs were cotreated with anti‐CXCR1, anti‐CXCR4, and anti‐CCL2 Abs. MSC migration toward lung cancer cells (both A549 and H1975) diminished after the blocking of CXCR1 or CXCR4. Data represent mean ± SEM (*n* = 10), and the *p* value reference was CTL.

The identification of MSCs with maximal homing ability toward lung tumors is a prerequisite to its use in conjugated nanodrugs. Different lineages of MSCs possess different distributions and types of chemokine receptors.^20^ For example, specific chemokine receptor showed selective responses toward various physiological environments (i.e., the inflammatory microenvironment of the body and chemotactic signals from damaged sites).^12^ BM‐MSC phenotypes were verified by positive CDs (i.e., 73, 90, and 105) and negative CDs (i.e., 34 and 45) (Figure S1, Supporting Information). In this study, BM‐MSCs demonstrated the best ability to target lung cancer cells among the other cancer cells (i.e., breast and brain cancer) (**Figure** **1**a).

To verify lung tumor tropism in vivo, A549 firefly luciferase (Fluc)–red fluorescence protein (RFP)‐bearing mice were confirmed via bioluminescence imaging (BLI) before MSC Fluc–green fluorescence protein (GFP) injection into the tail vein (Figure 1b). BLI signals showed that lung targeting of intravenously injected MSC_Fluc‐GFP_ was only significant in lung tumor‐bearing mice (i.e., A549), whereas no notable BLI signals of MSC_Fluc–GFP_ were detected in lung tumor‐free mice (Figure 1b). Furthermore, fluorescence‐activated cell sorting (FACS) analysis demonstrated threefold more MSC_Fluc–GFP_ in the lung tissues of lung tumor‐bearing mice than those of mice without lung tumors (Figure 1c and Figure S2a, Supporting Information).

To identify the mechanism of selective migration of BM‐MSCs toward lung cancer cells (H1975 and A549), chemokines from lung cancer cells were analyzed (Figure 1d–f and Figure S2b, Supporting Information). Selected chemokines such as CXCL12, IL‐8, and MCP‐1 were known as the major chemokines secreted from lung cancer cells.^21^ In this study, CXCL12 and IL‐8 were notably increased by A549 or H1975 when co‐cultured with MSCs (Figure 1d). Furthermore, the chemokines CXCL‐12 and IL‐8 from lung cancer cells strongly activated the associated chemokine receptors of MSCs, such as CXCR4 (CXCL12) and CXCR1 (IL‐8) in the presence of A549 and H1975 (Figure 1e). To identify the influence of CXCR1 and CXCR4 on MSC migration toward lung cancers, MSCs were treated with anti‐CXCR1 and anti‐CXCR4 Abs in the coculture system (i.e., membrane filter separation condition) (Figure 1f). MSC migration toward lung cancer cells (both A549 and H1975) was significantly diminished by Ab blocking (both CXCR1 and CXCR4) (Figure 1f). Simultaneous blocking of CXCR1 and CXCR4 significantly inhibited the MSC migration toward lung cancer compared with single Ab blocking (Figure 1f). However, blocking CCL2 (anti‐MCP‐1 Ab) hardly affected MSC migration toward lung cancer (Figure 1f and Figure S2b, Supporting Information).

### 2.2 Optimizing MSC–Nanodrug Conjugation

Traditionally, genetically engineered MSCs are introduced to increase anti‐cancer efficacy, such as by the secretion of therapeutic proteins (i.e., TNF‐related apoptosis‐inducing ligand (TRAIL), IFN‐α, and IFN‐β)^22^ or expression of suicide‐inducing enzymes (i.e., HSV‐tk and cytosine deaminase (CD)).^15^, ^23^ An alternative approach is to conjugate drugs or to facilitate intracellular drug loading into MSCs. However, nonoptimized drug conjugation or conventional intracellular drug loading into MSCs can diminish the intrinsic functionality of MSCs by reducing their homing ability, increasing self‐apoptosis, causing uncontrollable differentiation, and triggering unexpected tumorigenesis initiation of MSCs (with or without interaction with cancer cells).^24^

**Figure 2:**
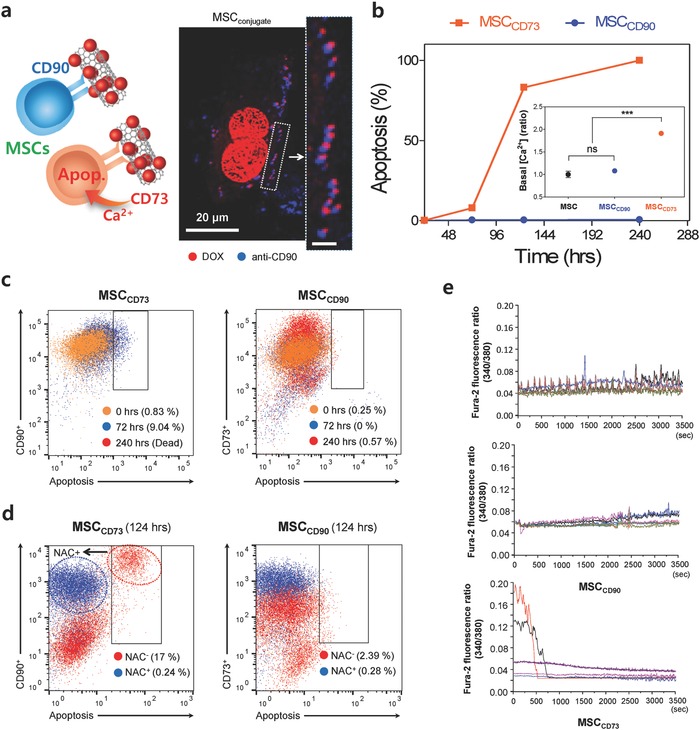
Optimizing MSC–nanodrug conjugation. a) Schematic shows MSC conjugation using CD90 or CD73. Representative confocal image shows adjacent localization (anti‐CD90 Ab, blue) and the nanodrug (red). The inset image shows a magnified view of the enclosed box. All bar is 20 µm. b) Influence of CD conjugation on MSC apoptosis and MSC intracellular calcium concentration ([Ca^2+^]_i_). When conjugated with CD90, apoptosis was 0.386%, 0.402%, and 0.57% at 72, 144, and 240 h, respectively. After 48 h, CD73 conjugation increased basal [Ca^2+^]_i_, whereas CD90 conjugation did not. Data represent mean ± SEM (*n* = 3). c) Time‐dependent apoptosis of MSC_CD73_ and MSC_CD90_. Apoptotic MSC cells were identified by BV421‐labeled annexin V. Note that CD73 conjugation (MSC_CD73_) induced complete apoptosis after 240 h. d) MSC conjugated with CD73 or CD90 nanodrug in the presence or absence of 100 × 10^−6^ m of NAC at 124 h. Note that the apoptotic response (17%) from MSC_CD73_ entirely disappeared with NAC (0.24%) and, thus, indicated that the apoptosis of MSC_CD73_ was triggered by ROS production. e) Changes in [Ca^2+^]_i_ were measured with Fura‐2 fluorescence ratiometric analysis (340/380) in MSCs conjugated with either CD73 or CD90.

**Figure 3:**
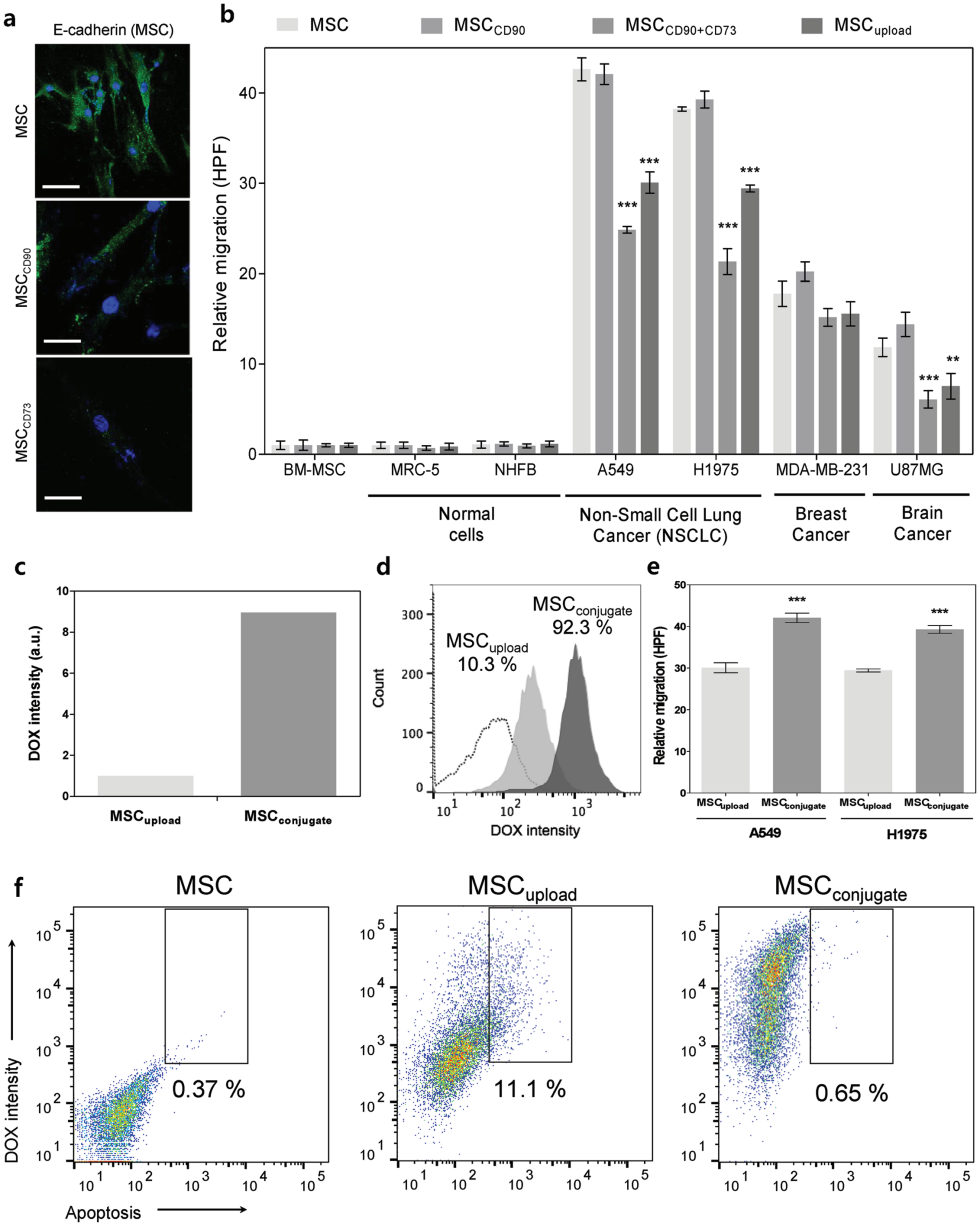
Optimizing MSC–nanodrug conjugation. a) Phenotypic expression of the plasma membrane marker E‐cadherin on the surface of MSCs imaged by confocal microscopy confirmed that CD73 conjugation induced cell membrane damage after 48 h. b) Impact of CD conjugation on MSC migration. A comparison in migration between conjugated MSCs (i.e., CD90 and CD90 + CD73) and intracellularly loaded MSCs toward normal and cancer cell lines. Data represent mean ± SEM (*n* = 10), and the *p* value reference was MSCs. c) Amount of DOX conjugated with MSC_conjugate_ and MSC_upload_. d) Percentage of MSC possessing DOX (for MSC_conjugate_ and MSC_upload_). e) Migration analysis of MSC_conjugate_ and MSC_upload_ to A549 and H1975 cells. Data represent mean ± SEM (*n* = 10). f) FACS analysis of the apoptosis of MSC, MSC_upload_, and MSC_conjugate_ after 24 h of nanodrug treatment (for reference, 1 × 10^4^ single cell events were acquired).

In this study, the functional stability of nanodrug‐conjugated MSCs with carbon nanotube (CNT)–Doxorubicin (DOX) through CD73 (MSC_CD73_), CD90 (MSC_CD90_: MSC_conjugate_), or by intracellular loading (MSC_upload_) was compared. MSC–nanodrug conjugation was systematically optimized based on the results from optimum incubation time and MSC viability (Figure S3a–e, Supporting Information). The exact amount of conjugated DOX on the membrane of BM‐MSCs was extrapolated considering quenching effect (Figure S3f, Supporting Information). Before comparing the changes in MSC homing ability of MSC_conjugate_ and MSC_upload_, selecting the appropriate CD–nanodrug conjugation on MSCs is essential. The examined lung cancer cells (i.e., H1975) exhibited negative for CD73 and CD90 among the positive MSC CDs (see Figure S1 in the Supporting Information), and, thus, these CDs were suitable for nanodrug conjugation by means of corresponding anti‐CD receptors (**Figure** **2**a). Confocal imaging confirmed the stable conjugation of DOX on the membranes of MSCs through anti‐CD90 or anti‐CD73 mobilization after 24 h (Figure 2a). The amount of drug (DOX) attached on the MSC membrane through either CD90 (MSC_CD90_) or CD73 (MSC_CD73_) was comparable, and the simultaneous conjugation of CD90 and CD73 (MSC_CD90+CD73_) increased the attached drug amount by about 40% compared to the singular conjugation of either CDs (i.e., CD73 or CD90) (Figure S3c, Supporting Information). However, the apoptosis of MSC induced by CD conjugation significantly differed depending on the CD type. When conjugated with CD90, MSC apoptosis was negligible (i.e., 0.386%, 0.402%, and 0.57% at 72, 144, and 240 h, respectively) (Figure 2b). In contrast, conjugation with CD73 drastically increased MSC apoptosis (i.e., 7.972%, 83.1%, and 100% at 72, 144, and 240 h, respectively) (Figure 2b). CD73 blocking by anti‐CD73 Ab caused greater apoptosis of MSCs after 72 h (Figure 2b) and, thus, CD73 conjugation is not appropriate in terms of MSC viability. To understand why CD73 conjugation induces complete apoptosis after 240 h, the generation of reactive oxygen species (ROS) and calcium influx were studied (Figure 2c–e). Most apoptotic signals from MSCs with CD73 conjugation disappeared after treatment with the ROS scavenger *N*‐acetylcysteine (NAC+) (Figure 2d). Furthermore, increased intracellular calcium concentration ([Ca^2+^]_i_) was observed in MSCs after CD73 conjugation (Figure 2e). Increased calcium ion overload in the MSC cytosol can incite strong activation of apoptosis and can produce highly toxic proteolytic enzymes.^25^ Immunostaining analysis of the plasma membrane marker E‐cadherin also verified that CD73 conjugation induced greater damage on cell adhesion‐type membranes (**Figure** **3**a) than CD90 conjugation. As such, inhibiting the function of CD73 through anti‐CD73 conjugation inhibited cell to cell adhesion, and triggered ROS production with massive intracellular Ca^2+^ overload that, consequently, induced cell death. These results indicate that CD73 plays a more significant role than CD90 in MSC survival, proliferation and differentiation,^26^, ^27^, ^28^ and, accordingly, CD90 is a better strategy for nanodrug conjugation.

Changes in homing ability after the nanodrug conjugation of MSC_CD73+CD90_, MSC_CD73_, and MSC_CD90_ suggested that MSC_CD73_ (singly or as MSC_CD73+CD90_) significantly diminished the targeting ability of MSCs to lung cancer cells (H1975) compared to MSC_CD90_ (Figure 3b). The homing ability of MSC_CD90_ was identical to that of untreated MSCs and, thus, MSC_CD90_ with the optimal amount of anti‐cancer drug attachment did not downregulate MSC homing ability to lung cancer cells (both A549 and H1975) (Figure 3b).

### 2.3 Drug Stability, Homing, and Self‐Apoptosis of MSC_conjugate_ and MSC_upload_

Before discussing anti‐cancer efficacy against lung tumor by conveying the anti‐cancer nanodrug through MSCs, the comparison of the drug stability of MSC_conjugate_ and MSC_upload_ is meaningful. FACS analysis showed that MSC_conjugate_ possessed nine times more DOX than MSC_upload_ (after 2 h of interaction with 1 µg mL^−1^ of DOX) (Figure 3c,d) at the same cell population (i.e., 10^4^ cells). The increased percentage of MSCs bearing the anti‐cancer drug not only boosts its anticancer efficacy (i.e., the amount of attached drug on MSC), but also reduces the dose of MSCs (i.e., the number of cells) required in clinical therapeutic applications.

The lung cancer homing abilities of MSC_conjugate_ and MSC_upload_ were also compared (Figure 3e). The homing ability of MSC_conjugate_ to lung cancer cells was similar to that of unmodified MSCs, whereas the lung cancer homing ability of MSC_upload_ was diminished. MSC apoptosis induced by nanodrug conjugation and nanodrug loading suggested that MSC_upload_ elevated apoptosis (Figure 3f). However, MSC_conjugate_ showed negligible apoptosis after 24 h (Figure 3f).

### 2.4 Apoptosis of Lung Cancer Cells Co‐cultured with MSC_conjugate_ or MSC_upload_

**Figure 4:**
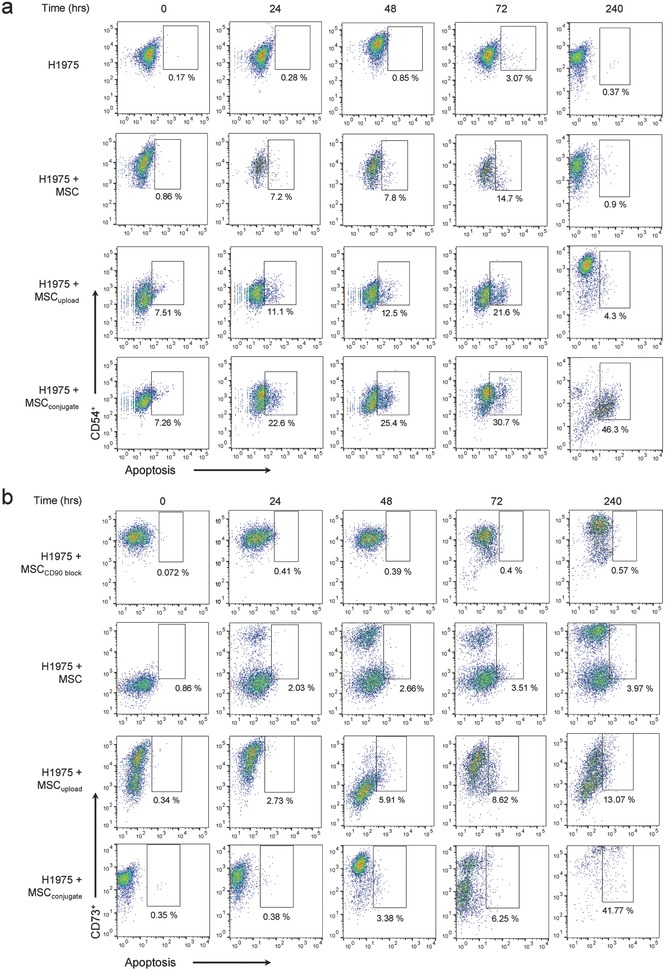
FACS analysis of H1975 and MSC apoptosis on co‐culture conditions. a) Time‐dependent FACS analysis of H1975 apoptosis when co‐cultured with MSCs, MSC_upload_, and MSC_conjugate_. Apoptotic H1975 cells were identified by BV421‐labeled annexin V. During the initial 72 h, apoptosis of lung cancer cells co‐cultured with MSCs steadily increased (i.e., MSC_conjugate_, 30.7%; MSC_upload_, 21.6%; and MSCs, 14.7%). However, after 240 h, lung cancer apoptosis decreased to negligible levels for MSC_upload_ (4.3%) and MSCs (0.9%), whereas a continued increased in lung cancer apoptosis was observed with MSC_conjugate_ (46.3%). b) Time‐dependent FACS analysis of MSC apoptosis when co‐cultured with H1975 cells was identified by BV421‐labeled annexin V.

**Figure 5:**
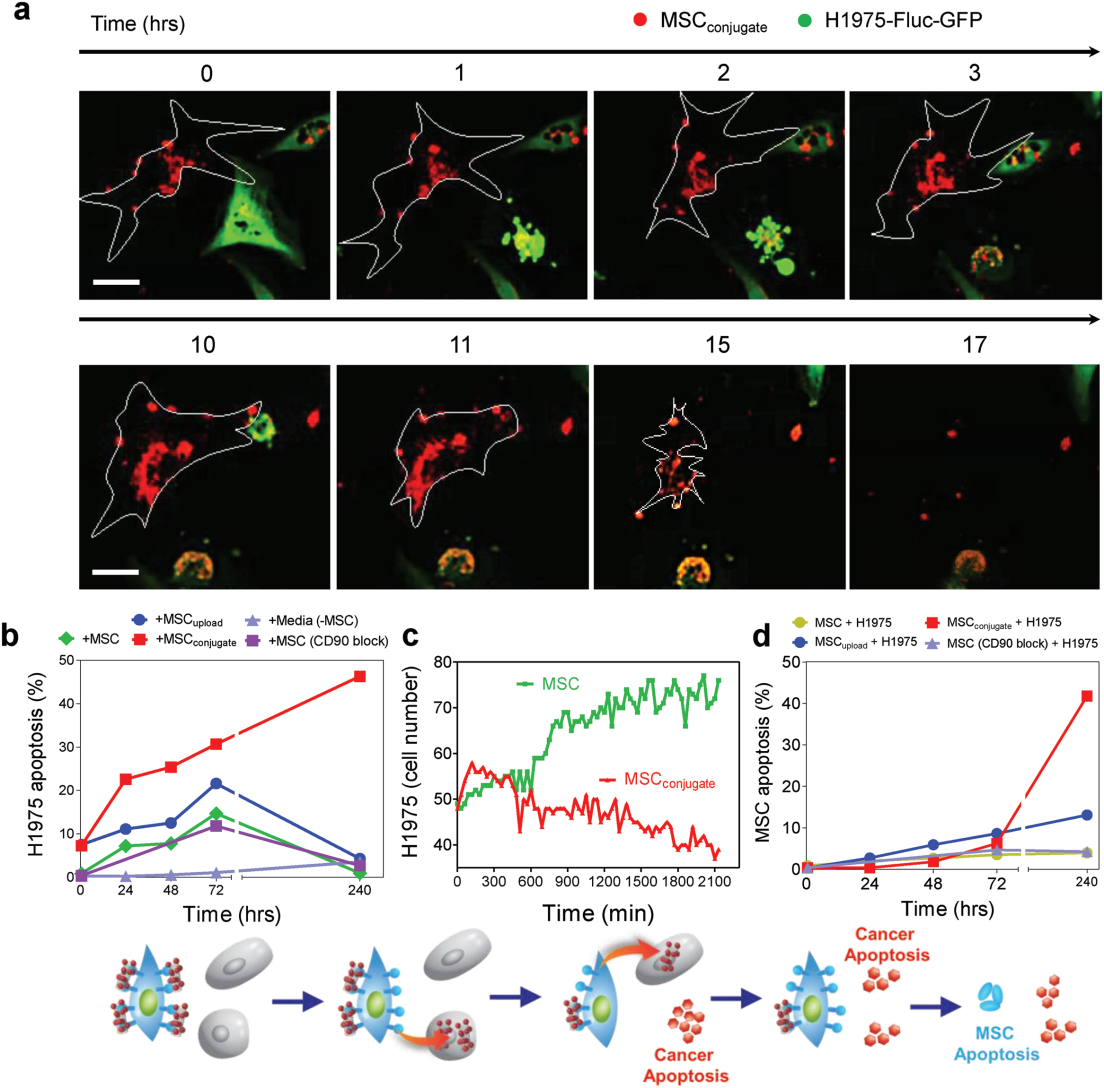
Mutual apoptosis of MSCs, MSC_upload_, and MSC_conjugate_ after lung cancer interaction. a) Select live cell images obtained by confocal quantitative image cytometer showing the apoptosis of H1975 cells near MSC_conjugate_ within 3 h of co‐culturing. All bars are 50 µm. b) Time‐dependent apoptosis profiles of H1975 cells co‐cultured with MSCs, MSC_upload_, and MSC_conjugate_. c) Time‐dependent H1975 population analysis of H1975 lung cancer cells when co‐cultured with MSC (green) and MSC_conjugate_ (red) was counted by confocal quantitative image cytometer. d) Time‐dependent apoptosis of MSC showed that more than 40% of MSC_conjugate_ entered the apoptotic stage after 240 h, whereas other types of MSCs (i.e., MSCs and MSC_upload_) exhibited no notable changes in apoptosis after 72 h.

For co‐cultured FACS analysis, selection of opposite CDs (i.e., positive/negative CD for MSCs but negative/positive CD for lung cancer cells) is essential. In this study, H1975 tested entirely positive (99.4%) for CD54 while A549 showed only partially positive (37.8%) results (see Figure S1, Supporting Information). Thus, only H1975 was selected as standard lung cancer cells when co‐cultured with MSC. Apoptosis of lung cancer cells co‐cultured with MSCs was analyzed for up to 240 h (i.e., 10 d). Interestingly, short‐term (0–72 h) and long‐term (72–240 h) apoptosis of lung cancer cells exhibited different trends. The apoptosis of lung cancer cells (H1975) when co‐cultured with MSCs and MSC_upload_ steadily increased until 72 h (**Figures** **4**a and **5**b). However, after 240 h, the apoptosis of H1975 co‐cultured with MSCs and MSC_upload_ returned to negligible amounts (Figures 4a and 5b). This indicated that MSCs and MSC_upload_ have no additional inhibitory influence on lung cancer survival under long‐term culture conditions. In contrast, MSC_conjugate_ significantly accelerated the apoptosis of lung cancer cells even after 10 d (Figures 4a and 5b). Specifically, more than 46% of lung cancer cells entered the apoptotic stage after 10 d when co‐cultured with MSC_conjugate_ (Figures 4a and 5b). As such, only MSC_conjugate_ elevated long‐term (≈240 h) lung cancer apoptosis, as compared to MSC and MSC_upload_. Selected live cell images and movies obtained by confocal quantitative image cytometer clearly showed triggered apoptosis for H1975 cells near MSC_conjugate_ within 3 h (Figure 5a and Movie 1 and 2, Supporting Information). However, normal cells (NHFB) did not exhibit any notable apoptotic signals in the presence of MSCs (Figure S4, Supporting Information). Live confocal quantitative image cytometer analysis clearly showed decrement in H1975 population when co‐cultured with MSC_conjugate_, whereas cell population of H1975 was slightly increased when co‐cultured with MSC (Figure 5c and Movie 3, Supporting Information).

In terms of MSC apoptosis, no significant changes were observed until 72 h for any of the tested MSCs (i.e., MSC, MSC_upload_, and MSC_conjugate_) when co‐cultured with H1975 (Figures 4b and 5d). However, after 10 d, more than 40% of MSC_conjugate_ entered the apoptotic phase, whereas other types of MSCs (MSC_upload_) showed no changes after 240 h (Figures 4b and 5d). Given that the apoptosis of MSC_conjugate_ that was not co‐cultured with lung cancer cells recorded less than 0.5% apoptosis after 240 h (Figures 4b and 5d), the observed increase in apoptosis was clearly induced by interaction with lung cancer cells (Figure 5d). In terms of mutual apoptosis for both lung cancer and MSC_conjugate_ after 240 h, the secretion of chemokines from dead cancer cells may, in turn, damage MSC_conjugate_, establishing a mutually destructive effect. The live cell images obtained by confocal analysis captured the mutual apoptosis of MSC_conjugate_, which occurs within a few hours of H1975 apoptosis (Figure 5a and Movie 1‐3, Supporting Information). Importantly, no significant tumorigenesis markers (i.e., CD31 and CD34) were found in MSC, MSC_upload_, or MSC_conjugate_ after 10 d of co‐culture with H1975 (Figure S5a–c, Supporting Information).

### 2.5 Cross Drug Delivery from MSCs to Lung Cancer Cells

**Figure 6:**
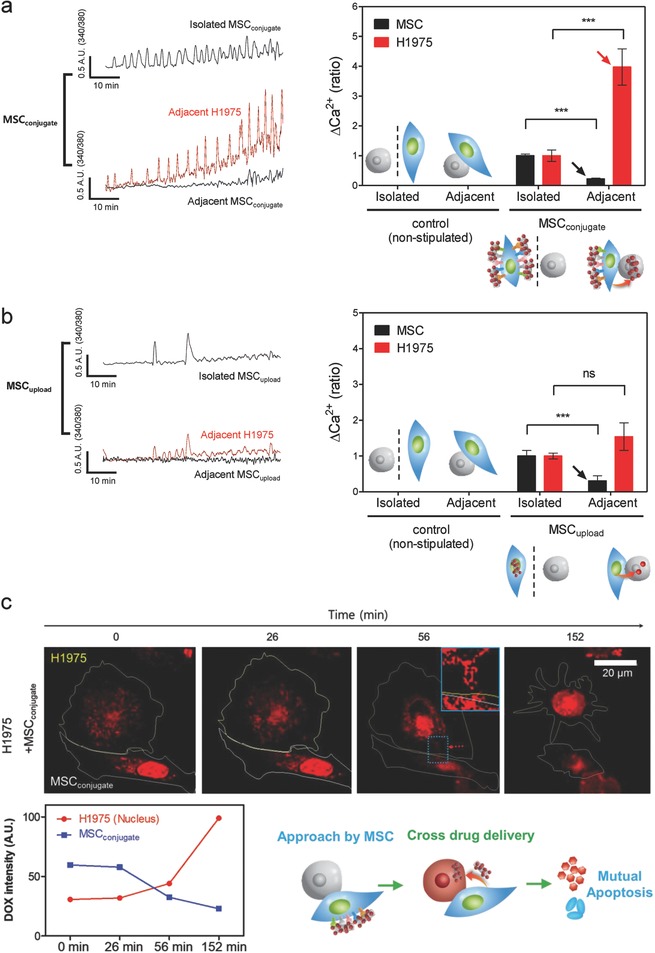
Intercellular drug delivery from MSCs to lung cancer cells. a,b) Analysis of [Ca^2+^]_i_ signals from H1975 and MSCs when co‐cultured with (a) MSC_conjugate_ or (b) MSC_upload_. Traces were separated between adjacent (i.e., near H1975 cells) and isolated MSCs (black) and H1975 cells (red). Intracellular calcium levels of H1975 cells when co‐cultured with MSC_conjugate_ increased significantly compared to that of MSC_upload_. Slight [Ca^2+^]_i_ signals for both isolated MSC_conjugate_ and isolated MSC_upload_ disappeared when co‐cultured with H1975. Data represent mean ± SEM (*n = 8*) ***p < 0.001 versus isolated MSC or isolated H1975. c) Select live cell images obtained by confocal analysis show intercellular drug (DOX) transfer (from MSC_conjugate_ to H1975) within 30 min. The time‐dependent trends in DOX intensity for MSC_conjugate_ and H1975 cells (bottom) show opposite patterns, and, after 2 h of drug transfer, H1975 cells exhibited apoptotic morphologies. All bars are 20 µm.

Intracellular Ca^2+^, as an important secondary messenger, regulates various cellular functions, including gene expression, metabolism, proliferation, apoptosis, contraction, and secretion.^27^ Ca^2+^ homeostasis is tightly regulated and critical in cellular physiology and pathophysiology and dysfunctional intracellular Ca^2+^ overload has been associated with cell apoptosis.^28^ In this study, no change in [Ca^2+^]_i_ was observed in isolated/adjacent MSCs or H1975 cells without nanodrug attachment (Figure S6, Supporting Information). It should be noted that isolated MSC_conjugate_ and MSC_upload_ (without lung cancer cells) exhibited small oscillatory [Ca^2+^]_i_ peaks owing to the presence of the nanodrug on MSCs (**Figure** **6**a,b).

Importantly, [Ca^2+^]_i_ of H1975 cells when co‐cultured with MSC_conjugate_ significantly increased and slight [Ca^2+^]_i_ signal in the adjacent MSCs was disappeared (Figure 6a). This demonstrates that DOX conjugated on MSCs successfully transferred to H1975. Interestingly, MSC_conjugate_ induced a stronger H1975 [Ca^2+^]_i_ amplitude and more oscillatory patterns compared to those of MSC_upload_ (Figure 6a,b). It was evident that the slight increase of [Ca^2+^]_i_ for both isolated MSC_conjugate_ and isolated MSC_upload_ disappeared when co‐cultured with H1975 (Figure 6a,b).

This represented the evidence that slight [Ca^2+^]_i_ signals evoked by attached nanodrug from MSCs directly moved to cancer cells. Previous studies have demonstrated several types of interaction mechanisms associated with MSCs and cancer cells through nanotubes, gap junctions, vesicles, and exosomes.^29^ Although the cross‐talk drug transfer mechanism between MSCs and tumor cells requires further investigation, this study clearly observed the transfer of [Ca^2+^]_i_ signals between MSCs and H1975, which might be related to a previously discussed interaction mechanism.^30^ Furthermore, selected live cell images obtained by confocal analysis clearly showed cross cellular drug (DOX) transfer (from MSC_conjugate_ to H1975) within 30 min (Figure 6c). The trend of time‐dependent DOX intensity between MSCs and H1975 showed opposite patterns, and after 2 h of drug transfer, H1975 exhibited apoptotic morphology (Figure 6c).

### 2.6 In Vivo Anti‐cancer Efficacy

**Figure 7:**
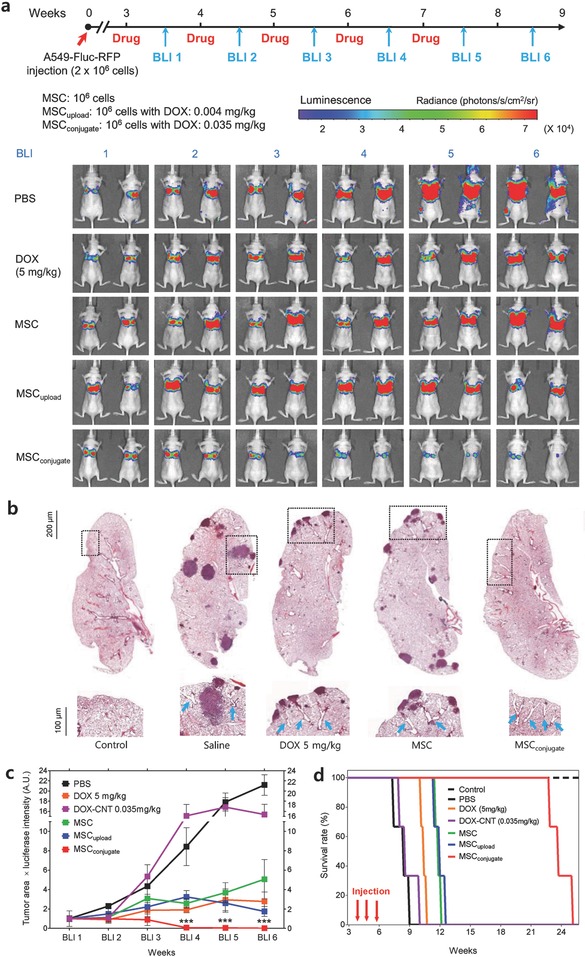
In vivo antitumor efficacy of MSC_conjugate_. a) BLI of luciferase expression of A549 lung tumor‐bearing mice after treatment with PBS, DOX (5 mg kg^−1^), MSCs (10^6^ cells), MSC_upload_, and MSC_conjugate_. b) H&E staining (at 100× magnification) of the lung from A549 tumor‐bearing mice after treatment with saline, DOX, MSCs, MSC_upload_, and MSC_conjugate_. Representative H&E staining shows the presence of tumor tissues. A magnified view of the enclosed box shows angiogenic blood vessels near tumor tissue. c) Time‐dependent luciferase expression in A549 lung tumor‐bearing mice after treatment with saline, DOX, DOX–CNT, MSCs, and MSC_conjugate_. Data represent mean ± SEM (*n* = 3). Quantitative analysis was based on the product of luciferase intensity and tumor area, as determined by BLI. d) Kaplan–Meier survival curve for mice with A549_Fluc–RFP_ lung tumors after the administration of saline, DOX, DOX–CNT, MSCs, and MSC_conjugate_ (*n* = 3). Arrows indicate time points of i.v. injections with tested drugs.

The percentage of lung tumor‐bearing mice by tail vein injection of H1975_Fluc–RFP_ (20%) was lower than that of A549_Fluc–RFP_ (70%) and, thus, only A549‐bearing lung tumor mice were used for the in vivo study. Three weeks after the tail vein injection of A549_Fluc–RFP_ cells, lung tumor‐bearing mice were sorted based on similar tumor size and signal intensity by BLI prior to the administration of the test drugs (DOX 5 mg kg^−1^, DOX–CNT 0.035 mg kg^−1^, MSCs, MSC_upload_, and MSC_conjugate_) (**Figure** **7**a and Figure S7a, Supporting Information). BLI was conducted to analyze the variation in luciferase intensity and the size of lung cancer tumors (Figure 7a,c and Figure S7a, Supporting Information). Significant decreases in luciferase intensity from the lung tumors were found in the MSC_conjugate_ group, whereas the DOX, DOX–CNT, MSC, and MSC_upload_ groups simply exhibited a delayed growth rate of lung tumors compared to the saline group phosphate buffered saline (PBS) (Figure 7a,c and Figure S7a, Supporting Information).

Hematoxylin and eosin (H&E) staining of lung tissue obtained from saline, DOX, DOX–CNT, and MSC groups identifies many sites of the tumor tissues and, importantly, many blood vessels were found near the tumor tissues, whereas no blood vessels were identified near the surface in the control lung tissues (Figure 7b and Figure S7b, Supporting Information). Interestingly, the mouse treated with MSC_conjugate_ showed an absence of lung tumor tissues, even though many blood vessels were identified near the surface in the lung tissues (Figure 7b). This indicates that MSC_conjugate_ successfully removed the lung tumor sites, as confirmed by BLI (Figure 7a,c).

A survival test showed a nearly twofold extension in survival time for groups treated with MSC_conjugate_ compared to that of MSC, MSC_upload_, DOX (5 mg kg^−1^), and DOX–CNT (0.035 mg kg^−1^) after three cycles of drug injection (Figure 7d). The results demonstrated that only MSC_conjugate_ removed the lung tumor, whereas the other treatment types (i.e., both DOX and MSCs only) merely delayed lung tumor growth. This confirmed that, among the agents examined in this study, only MSC_conjugate_ is effective as a therapeutic agent for treating lung tumors.

### 2.7 In Vivo Safety Evaluation

**Figure 8:**
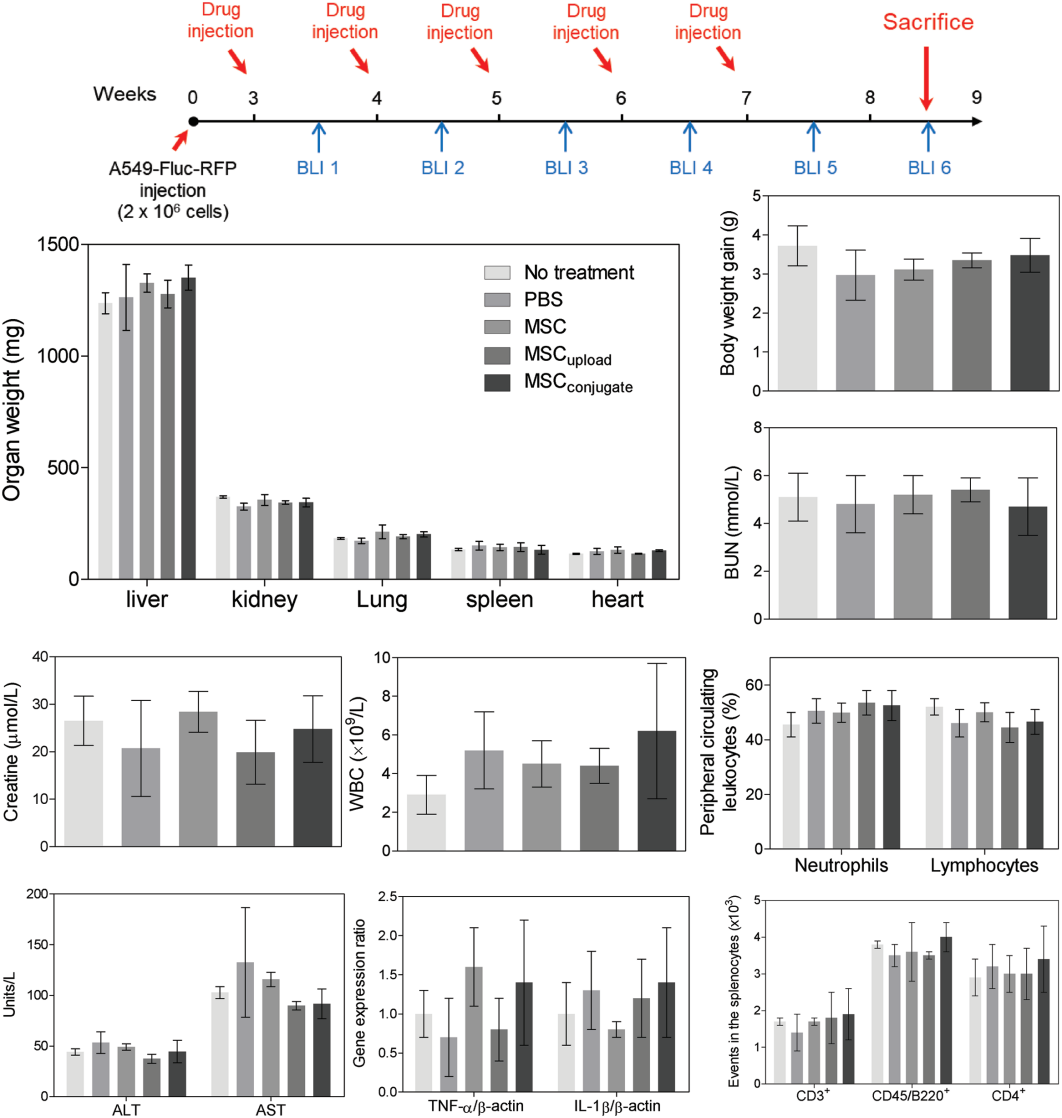
In vivo safety evaluation. BALB/c mice were sacrificed at 8 weeks (BLI 6) after A549_Fluc–RFP_ injection and showed no notable changes in weight (i.e., body and organ weight) or in general immunotoxicity profiles for MSC‐treated mice compared with nontreated controls. Abbreviations: ALT, enzymatic activity of alanine transaminase; AST, enzymatic activity of aspartate aminotransferase; BUN, blood urea nitrogen; WBC, white blood cells.

In vivo safety is a critical factor in the preclinical evaluation of groups intravenously injected with MSCs. In this study, no obvious weight loss was observed in the mice (**Figure** **8**), and no significant differences in organ weight, including the liver, kidney, heart, spleen, and lung, were observed at 8 weeks after A549_Fluc–RFP_ injection. Blood chemistry tests (i.e., alanine aminotransferase (ALT), aspartate aminotransferase (AST), creatinine, and blood urea nitrogen (BUN)) and peripheral circulating leukocyte count (i.e., white blood cells (WBC), lymphocytes, and neutrophils) revealed no changes in any groups compared to the control mouse (Figure 8). Although the cardiotoxicity of high DOX doses usually causes side effects,^31^ the H&E staining showed that the heart tissue remained intact (Figure S8, Supporting Information), indicating the cardiac safety of the tested MSCs and MSC_conjugate_.

To assess the extent of lung inflammation, gene expression levels of the proinflammatory cytokines, such as TNF‐α and IL‐1β, were measured via quantitative PCR (qPCR). No changes were observed (Figure 8). In addition to the absence of inflammatory signs in lung tissue (Figure S8, Supporting Information), the results implied that the anti‐cancer effect of MSC_conjugate_ did not arise from alteration of the lung microenvironment, but by direct targeting of the tumor cells. Sometimes, nanomaterials interfere with normal immune function without inducing liver and/or kidney toxicity.^32^ To examine the immunotoxicity stimulated by MSCs, splenic lymphocyte populations (e.g., CD3^+^ T cells, CD45R^+^ B cells, and CD3^+^CD4^+^ T helper cells) were analyzed. Again, MSCs, MSC_upload_, or MSC_conjugate_ did not induce any notable immunotoxicity (Figure 8).

In addition, it has been reported that CNT may induce pulmonary toxicity. However, no notable changes in lung weight and no notable inflammatory markers (e.g., TNF‐α and IL‐1β) were observed in this study even after five doses of nanodrug‐conjugated MSCs (Figure 8). The absence of pulmonary toxicity may stem from the low injection dose of CNTs (i.e., 0.1 mg kg^−1^ per 10^6^ MSCs). Furthermore, to identify the maximum tolerable dose of CNTs (as well as CNT–DOX conjugation), single and repeated exposure toxicity evaluations (e.g., weight changes in the lung) were performed (Figure S9, Supporting Information). Significant weight changes in the lung were observed in 5 mg kg^−1^ of CNT–DOX conjugation (Figure S9, Supporting Information). However, weight changes in the lung originated from the toxicity of CNT–DOX conjugation and not by CNTs alone because no notable weight changes in lung were observed for injection with 20 mg kg^−1^ of CNTs (which corresponded to the amount of CNT in 5 mg kg^−1^ of CNT–DOX conjugation) for both single and repeated exposure toxicity evaluations (Figure S9, Supporting Information). Taken together, no pulmonary toxicity was induced by nanodrug‐conjugated MSC due to the low concentration of DOX and CNTs (i.e., 0.035 and 0.1 mg kg^−1^, respectively) conjugated on MSC (10^6^ cells) (Figure 8).

## 3 Conclusion

The stem cell therapy market already possesses several FDA‐approved products. Authorized therapies include the treatment for autoimmune diseases and Crohn's disease (Prochymal, Osiris Therapeutics), cardiac disease (Hearticellgram, Pharmicell), severe limbal stem cell deficiency (Holoclar, Chiesi Farmaceutici), and knee cartilage defects (Caritstem, MEDIPOST). Despite its increasing clinical significance, stem cell therapy for the treatment of malignant tumors has not yet been authorized because previous stem cell therapy required genetic modification of MSC to increase cancer apoptotic ability. Furthermore, the clinical approval of anti‐cancer nanodrugs, such as liposomal and PEGylated drugs, has been extremely limited due to safety issues, including body clearance and materials degradation. Inorganic nanodrugs, such as gold, silica, and carbon‐based nanomaterials, encountered similar safety concerns (i.e., low clearance and nondegradability). For these reasons, the use of nanomaterials requires achieving clinical efficacy with extremely small amounts of nanomaterials (i.e., with low drug concentrations or increased enzymatic activity). However, we need to reevaluate the clinical use of nanomaterials within limited conditions (i.e., ultra‐low concentration) if there is no superior substitute to nanomaterials in terms of drug delivery vehicles. A previous study focused on silica‐nanorattle nanoparticles conjugated on the MSC membrane, which successfully demonstrated membrane‐conjugated nanodrug delivery in the skin‐xenograft mice model.^19^ However, without a thorough understanding and optimization of nanodrug–MSC conjugation and without success on deep tissue tumor model, such as lung, brain, and pancreas, clinical application will be difficult. In this aspect, covalently conjugated nanodrugs on the membrane of stem cells achieve anti‐cancer efficacy on deep tissue tumor model at ultralow concentrations and, thus, can overcome the side effects associated with toxicity. The dose of the stem cell‐conjugated anti‐cancer nanodrug presented in this study was extremely low (DOX: 0.035 mg kg^−1^ and CNT: 0.1 mg kg^−1^), thanks to efficiently coated anti‐cancer nanodrugs on the membrane of MSCs without diminishing the innate functionality of stem cells (i.e., homing ability).

In conclusion, this study presented nanodrug‐conjugated MSCs that successfully harnessed the innate homing ability of BM‐MSCs to lung tumor for the improvement of anti‐cancer efficacy. Importantly, this strategy circumvents the need for genetic modification and the exact targeting of MSCs to lung tumors minimizes the dose of the anti‐cancer drug required by several hundred times compared to conventional chemotherapy. The present findings might be applicable not only to lung cancer treatment, but also to the treatment of other types of cancer that requires exact drug targeting. For example, by switching the type of conjugated drug and/or the nanomaterial, this strategy can be applied for the treatment of other malignant tumors (e.g., brain and pancreas) and to autoimmune‐related inflammatory diseases (e.g., arthritis). As such, this study introduced a novel platform technology of stem cell membrane‐conjugated therapies that is potentially applicable for the treatment of a broad spectrum of diseases which require a high targeting ability.

## 4 Experimental Section

*Cell Culture*: Human BM‐MSCs (PT‐2501, Lonza) were cultured in MSC medium (MSCBM: PT‐3238, Lonza) with supplements (PT‐4105, Lonza). The human NSCLC cell line H1975 (CRL‐5908, ATCC) was maintained in Roswell Park Memorial Institute 1640 medium (RPMI 1640: 11875‐093, Gibco) supplemented with 10% fetal bovine serum (FBS: 26140, Gibco) and 1% antibiotics (10378016, Gibco). The human NSCLC cell line A549 (CCL‐185, ATCC), human breast cancer cell line MDA‐MB‐231 (HTB‐26, ATCC), human fibroblast cell line NHFB (CC‐2511, Clonetics), and human glioma cell line U87MG (HTB‐14, ATCC) were maintained in high‐glucose Dulbecco's modified Eagle's medium (11995‐065, Gibco) supplemented with 10% FBS (16000‐044, Gibco) and 1% antibiotics (10378016, Gibco). Normal lung fibroblast cells, MRC‐5 (CCL‐171, ATCC), were maintained in Eagle's minimum essential medium (30–2003, ATCC) supplemented with 10% FBS (26140, Gibco) and 1% antibiotics. All cultures were maintained in 5% CO_2_ at 37 °C.

*In vitro Homing Analysis*: One day before the assay, BM‐MSCs, normal cells, and cancer cells were seeded at 1 × 10^5^ cells in 24‐well plates (CLS3527, Corning) and cultured overnight in 500 µL of complete medium. BM‐MSCs (1 × 10^5^) were then plated onto the top side of transwell chambers (#3422, polycarbonate membrane, 24‐well format, 8‐µm pore size, Corning). After 6 h, the cells spreading on the bottom sides of the membrane were fixed with 100% methanol for 1 min and stained with 4′,6‐diamidino‐2‐phenylindole (DAPI) (D1306, Molecular Probes). Ten fields per sample were measured at 400× magnification (high‐power field: HPF). The number of MSCs homing to cancer cells through the membrane pores was counted, and the mean number of cells/HPF was determined.

*Flow Cytometry*: For FACS analysis, all cells (both single‐culture and co‐culture conditions) were detached from wells using 0.25% trypsin (1 mL, Gibco) for 5 min at 37 °C and mechanically dissociated to a single‐cell suspension after the addition of FBS (10%, 0.5 mL). Detached cells were passed through a 40 mm cell strainer (93070, SPL), centrifuged, washed with PBS supplemented with 2% of FBS, and incubated for 30 min at room temperature (RT) with tested Abs in PBS containing 2% FBS. After a final washing step, the cells were resuspended in 500 mL PBS (supplemented with 10% FBS) and analyzed on a flow cytometer (BD LSRII, BD Biosciences). Ten thousand events were acquired per sample, and data were displayed on logarithmic scales. Forward and side light scatter signals were used to exclude dead cells and debris. Data were analyzed using the FlowJo software (Ver. 10.1, FlowJo, LLC). MSCs and H1975 cells in the absence of appropriate Abs conjugated to fluorescent dye were used to determine the background signal (0.1%) and determine gate boundaries.

To examine the homing ability of BM‐MSCs to migrate toward lung tumor tissues in vivo, tail veins of mice with or without A549 FLuc–RFP lung tumor were injected with BM‐ MSCs Fluc–GFP (2 × 10^6^ cells per mice), and after 4 d, the mice were sacrificed. Lungs from tumor‐bearing and tumor‐free mice were homogenized individually in 7 mL of collagenase type II (17101015, Gibco) with 1 mg mL^−1^ in MEM (Gibco) and incubated for 2 h in a CO_2_ incubator at 37 °C. Subsequently, the enzyme‐digested lung tissues were washed in PBS and analyzed via FACS.

To characterize surface CD expression of BM‐MSCs and H1975 cancer cells, detached cells were incubated for 30 min at RT with fluorescein isothiocyanate (FITC)‐conjugated or phycoerythrin (PE)‐conjugated Abs against CD34 (555821, BD Pharmingen), CD73 (550257, BD Pharmingen), CD90 (555596, BD Pharmingen), CD45 (555483, Becton‐Dickinson), and CD105 (323206, Biolegend). For determining the H1975 lung cancer‐specific marker, H1975 cells were analyzed by CD73 (550257, BD Pharmingen) and CD90 (555596, BD Pharmingen) Abs.

To examine the apoptosis of nanodrug‐conjugated or loaded BM‐MSCs, H1975 cells (4 × 10^5^) were cultured in a 10 cm culture dish for 24 h and co‐cultured with nanodrug‐conjugated or nanodrug‐loaded BM‐MSCs (5 × 10^5^) for 1–240 h. A total of 1 × 10^4^ cells per tested co‐culture were then incubated for 30 min at RT with anti‐CD73 (1:100, 561258, BD Pharmingen) or anti‐CD90 Abs (1:100, 562556, BD Pharmingen) with annexin V (1:100, A35122, Molecular Probes), and analyzed them using FACS.

To determine the tumorigenesis of nanodrug‐conjugated or nanodrug‐loaded BM‐MSCs, H1975 cells (4 × 10^5^) were cultured in 10 cm culture dishes and mixed with nanodrug‐conjugated or nanodrug‐loaded BM‐MSCs (4 × 10^5^) in 10 cm culture dishes for 10 d. Detached co‐cultured cells were incubated for 30 min at RT with anti‐CD31 (1:100, 564089, BD Horizon) and anti‐CD34 (1:100, 562577, BD Biosciences) Abs, and analyzed using FACS.

Flow cytometric analysis of lymphoid populations was performed on freshly isolated splenic cellular extracts. Cells were stained using anti‐mouse CD3‐FITC (total T cells), anti‐mouse CD45R/B220‐PE (B cells), and anti‐mouse CD4‐PerCP‐Cy5.5 (T helper cells). Abs were added to samples, gently vortexed, and incubated for 30 min at 4 °C. The fluorescence intensity was detected via FACS.

*Covalent Anti‐CD90–DOX–Carbon Nanotube Conjugates: N*‐ethyl‐*N*′‐(3‐dimethylaminopropyl)carbodiimide hydrochloride‐linked multiwalled CNTs were prepared,^33^ mixed with 20 µg mL^−1^ of anti‐CD90 Abs (ab23894, Abcam), and rotated at 4 °C for 2 h in MES (pH 6). Then, DOX was added (44583, Sigma) at a weight ratio of 1:1 (DOX:CNT) in MES buffer (pH maintained at 6.0). The mixture was rotated at 4 °C overnight. The anti‐CD90–CNT–DOX suspension in MES was centrifuged and filtered (Amicon YM‐100K, 4000 *g*) more than thrice to remove the unconjugated drug. Finally, anti‐CD90–CNT–DOX was dispersed and stored in PBS (10010‐023, Gibco) at 4 °C. To measure the concentration of DOX and CNTs, UV–vis adsorption spectroscopy (Libra S50, Biochrom) was performed at a wavelength of 490 nm. The weight percentage of covalently linked DOX on CNTs was determined by measuring the difference in absorbance signal intensities at 490 nm and extrapolation based on linear standard curves from the concentration of DOX and CNTs, respectively.

*MSC–Nanodrug Conjugates*: To conjugate the nanodrug with BM‐MSCs, 5 × 10^5^ cells were treated with a final concentration of 1 µg mL^−1^ DOX in anti‐CD90–CNT–DOX (volume: 7–10 µL, depending on percent nanodrug conjugation) for 2 h with occasional shaking at 37 °C. Then, BM‐MSCs were collected and thoroughly washed twice. The amount of DOX on BM‐MSCs was measured with a fluorescent microplate‐reader (Victor X3, PerkinElmer) and then quantitatively analyzed based on the standard curves of DOX (Figure S4f, Supporting Information).

*Confocal Analysis*: To observe the MSC–nanodrug conjugation, BM‐MSCs were cultured overnight on poly‐d‐lysine‐coated coverslips in 24‐well plates. A final concentration of 1 µg mL^−1^ DOX in 10 µL of anti‐CD90–CNT–DOX was used to treat the BM‐MSCs and incubated for 2 h. The BM‐MSCs were then washed with PBS thrice and fixed with 2% paraformaldehyde in medium overnight at 4 °C. After a blocking period of 2 h with 1% BSA in PBS, the cells were incubated with Alexa Fluor 405 goat anti‐rabbit IgG (H+L) (1:200 dilution factor, A31556, Molecular Probes) to visualize anti‐CD90 Abs for 2 h at RT. To observe the expression of E‐cadherin on the surface of MSC_CD73_ and MSC_CD90_, MSCs were cultured overnight on poly‐d‐lysine‐coated coverslips in 24‐well plates. A final concentration of 1 µg mL^−1^ DOX in 10 µL of anti‐CD90–CNT–DOX or anti‐CD73–CNT–DOX was used to treat the BM‐MSCs and incubated for 2 h and fixed with 2% paraformaldehyde. The fixed cells were incubated with mouse anti‐E‐cadherin antibody (13‐1900, Invitrogen) at RT for 1 h and then incubated with Alexa Fluor 488 goat anti‐rabbit IgG (H+L) (1:200 dilution factor, A31556, Molecular Probes) to visualize E‐cadherin on the MSC surface at RT for 1 h. Finally, cells were mounted, visualized using confocal microscopy (LSM700, Carl Zeiss), and analyzed using the ZEN software. To record live cell dynamics of MSC_conjugate_ with H1975 and MSC with H1975, live cell image system (CQ1, Yokogawa) was operated for 36 h after coculture of H1975 with MSC_conjugate_ (2 h of preincubation).

*Measuring DOX Amount of MSC_conjugate_ and MSC_upload_*: To compare DOX amounts in BM‐MSCs after loading or conjugating the nanodrug, FACS analysis was performed. BM‐MSCs (5 × 10^5^) were treated with anti‐CD90–CNT–DOX or CNT–DOX (1 µg mL^−1^ DOX) for 2 h in 10 cm dishes. Then, the cells were thoroughly washed and collected using trypsin. The DOX content of cells was analyzed using FACS with 488 nm excitation and 580 nm emission.

*MSC Functionality by Loading or Conjugation*: Comparative MSC migration ability after loading or conjugating the nanodrug with BM‐MSCs was performed using transwell chambers (polycarbonate membrane, 24‐well with 8 µm pore size, Corning). BM‐MSCs (5 × 10^5^) were treated with 7 µL CNT–DOX or 10 µL anti‐CD90–CNT–DOX (final DOX concentration of 1 µg mL^−1^) for 2 h. Nanodrug‐loaded or conjugated BM‐MSCs (5 × 10^4^) suspended in 200 µL medium were placed in the upper chamber of the transwell. The lower chamber was filled with 500 µL of conditioned medium with test BM‐MSCs, normal cells, and cancer cells. After 5‐h incubation, the number of DAPI‐stained cells migrating to the membrane was counted and analyzed.

*Intracellular Ca^2+^ concentration*: H1975 cells and BM‐MSCs were co‐cultured on cover glass, incubated with 4 × 10^−6^ m of Fura‐2 AM (F1201, Molecular Probes) in the presence of 0.05% Pluronic F‐127 (P3000MP, Molecular Probes) for 15 min in physiological salt solution (PSS) at RT in the dark, and washed with PSS for 10 min. The PSS contained 140 × 10^−3^ m NaCl, 5 × 10^−3^ m KCl, 1 × 10^−3^ m MgCl_2_, 1 × 10^−3^ m CaCl_2_, 10 × 10^−3^ m 4‐(2‐hydroxyethyl)‐1‐piperazineethanesulfonic acid (HEPES), and 10 × 10^−3^ m d‐glucose, titrated to pH 7.4. Changes in intracellular Ca^2+^ were measured based on the intensity of fluorescence with excitation wavelengths of 340 and 380 nm and an emission wavelength of 510 nm. All results were reported as the F340/F380 fluorescence ratio. Fluorescence was monitored with a charge‐coupled device camera (Retiga 6000 CCD camera, Photometrics) attached in an inverted microscope (IX71, Olympus) and analyzed with MetaFluor software (version 7.8, Molecular Devices). Fluorescence images were obtained at 3‐s intervals.

Background fluorescence was subtracted from the raw background signals at each excitation wavelength. The number of cells used in ΔCa^2+^ analysis was counted using Integrated Morphometry Analysis of MetaMorph software (version 7.8, Molecular Devices).

*Generation of MSC_Fluc–GFP_ and A549_Fluc–RFP_ Cells*: MSCs or A549 cells were seeded into 6‐well plates at a density of 10^5^ per well and allowed to attach overnight. The next day, the medium was changed to a mixture of 2 mL complete medium and 1 mL supernatant of lentivirus (10^5^ viral particles per well) encoding the Fluc and RFP gene (#GlowCell‐14b‐1, Biosettia) or green fluorescence protein (GFP) gene (#GlowCell‐16p‐1, Biosettia). After 24 h, the culture medium containing lentivirus was discarded, and the cells were cultured for another 72 h. Post transduction, the cells were cultured in medium supplemented with puromycin (1 mg mL^−1^) to eliminate the nontransduced cells. After initial selection, the cells were cultured in medium supplemented with 0.3 mg mL^−1^ puromycin for 1 month. Stably transfected MSC_Fluc–GFP_ and A549_Fluc–RFP_ cells were confirmed by bioluminescence imaging using 150 mg mL^−1^ d‐luciferin and an in vivo imaging system (IVIS, IVIS‐200, Xenogen).

*Bioluminescence Imaging*: BLI was used to measure luciferase activity by IVIS imaging optimized for high sensitivity. BLI was performed every week. Mice were anesthetized with isoflurane (≈3% in air) and then intraperitoneally injected with luciferin (150 mg kg^−1^, Xenogen) 40 min before imaging. The animals were then put in the light‐protected chamber of the IVIS and measured for 1 min. Regions of interest were captured using Living Image software (ver. 3.1, Xenogen). BLI was measured based on radiance as the average luminescence units detected on the surface of the animal per square centimeter per steradian (photons s^−1^ cm^−2^ sr^−1^).

*A549 Lung Tumor Model*: Female BALB/c nude mice (4–6 weeks old, weighing 16–20 g each) were purchased from Orient Bio (Seoul, Korea). All animal experiments were carried out under the regulation of the Care and Use of Laboratory Animals of Gachon University (IRB number: GCIRB2015‐83). To establish lung tumor‐bearing mice, 3 × 10^6^ A549_Fluc–RFP_ cells were suspended in 200 µL of PBS and intravenously injected into the tail vein of mice. To confirm the establishment of the A549_Fluc–RFP_ lung tumor model, mice were intraperitoneally injected with 150 mg kg^−1^ of d‐luciferin and imaged after 40 min with Xenogen IVIS to analyze the size of the tumor growth.

*In vivo antitumor efficacy*: Mice bearing lung tumors (abdominal bioluminescence greater than 1 × 10^7^ photons s^−1^) were randomly divided into six groups (*n* = 3). The tail vein of each mouse was injected intraperitoneally with MSC_conjugate_ (10^6^ cells, DOX: 0.035 mg kg^−1^, CNT: 0.08 mg kg^−1^), MSCs (10^6^ cells), DOX (5 mg kg^−1^), DOX–CNT (0.035 mg kg^−1^), and saline (in 200 µL PBS) every week for 5 weeks. The tumor size was monitored by measuring the intensity by IVIS every week.

*Peripheral Blood Sample Analyses*: At the end of the in vivo experimental period, blood samples were collected from the celiac artery. For the analysis of peripheral circulating blood cells, blood samples were placed in a labeled vial containing heparin (5 units per mL) and transported on ice for hematological analysis. Blood cells were automatically counted (Sysmex F‐820 Blood Counter, Toa Medical Electron). For serum, the blood was allowed to clot by leaving it undisturbed at RT; the clot was removed and centrifuged at 2000 *g* for 15 min at 4 °C, and the supernatant was retained. Levels of ALT, AST, and creatinine or BUN were measured using clinical chemistry reagent kits (HUMAN).

*qPCR*: To detect the expression of cytokines, qPCR was performed using the Dice thermal cycler (TP850, Takara) according to the manufacturer's protocol. At the end of the in vivo experimental period, the lung tissues were excised and total RNA was isolated using RNAiso Plus (#9108, Takara). First‐strand complementary DNA (cDNA) was synthesized using the reverse transcription premix (iNtRON Biotech). The reverse transcription conditions were 45 °C for 60 min and 95 °C for 5 min. Briefly, 2 µL of cDNA (100 ng), 1 µL each of sense and antisense primer solutions (0.4 × 10^−6^ m), 12.5 µL of SYBR Premix Ex Taq (Takara), and 8.5 µL of dH_2_O were mixed together to obtain a reaction mixture with a final volume of 25 µL in each reaction tube. The primers used for qPCR were β‐actin (F: 5′‐TAG ACT TCG AGC AGG AGA TG‐3′; R: 5′‐TTG ATC TTC ATG GTG CTA GG‐3′), TNF‐α (F: 5′‐GGC AGG TCT ACT TTG GAG TCA TTG C‐3′; R: 5′‐ACA TTC GAG GCT CCA GTG AAT TCG AAT TCG G‐3′), and IL‐1β (F: 5′‐ATA ACC TGC TGG TGT GTG AC‐3′; R: 5′‐AGG TGC TGA TGT ACC AGT TG‐3′). The normalization and quantification of mRNA expression were performed using TP850 software supplied by the manufacturer. The expression level of β‐actin was used for normalization.

*Single‐Cell Lymphocyte Populations*: At the end of the experiment, mice were euthanized with CO_2_, and the spleens were collected from each mouse. The spleen was aseptically excised and maintained in 10 mL of cold complete RPMI 1640 with 10% heat‐inactivated FBS and 1% antibiotics (10378016, Gibco). Monocellular suspensions were prepared using a Stomacher laboratory blender (Stomacher 80, Seward). The cell suspension was washed with RPMI medium. The cell pellets were resuspended in RPMI medium and incubated for 4 h in 5% CO_2_ at 37 °C. To collect lymphocytes, the suspended cells were transferred to a new tube separating macrophages and granulocytes. Viable cells were counted using a hemocytometer (Paul Marienfeld GmbH and Co. KG).

*Single and Repeated Exposure Toxicity Test*: Male and female Imprinting Control Region (ICR) mice (6 weeks old) were purchased from Dae‐Han Experimental Animal Center (Daejeon, Korea). Mice were randomly assigned to treatment cages and acclimated for 1 week. Mice had ad libitum access to standard rodent chow and filtered water. Throughout the study, the mice were housed in a laminar airflow room in a controlled environment (temperature, 22 ± 2 °C; relative humidity, 55 ± 5%; 12‐h/12‐h light/dark cycle). The care and treatment of the animals were in accordance with the guidelines established by the Public Health Service Policy on the Humane Care and Use of Laboratory Animals and were approved by the Institutional Animal Care and Use Committee (IRB #2016‐0151). Mice (*n* = 10 per group) were administered by intravenous injection with 200 µL of saline, DOX (5 mg kg^−1^), CNT (20 mg kg^−1^), CNT–DOX (DOX: 0.2 mg kg^−1^ and CNT: 0.8 mg kg^−1^), CNT–DOX (DOX: 1 mg kg^−1^ and CNT: 4 mg kg^−1^), CNT–DOX (DOX: 5 mg kg^−1^ and CNT: 20 mg kg^−1^) in the tail vein. For single exposure test, mice were injected with respective drugs single time and monitored during 2 weeks. For repeated exposure test, mice were injected with drugs twice a week (total four times) and monitored during 2 weeks. At the end of treatment period, mice were fasted overnight and euthanized by carbon dioxide. Trunk blood was collected, and various organs were aseptically excised and weighed.

*Histology*: Tissues isolated from mice were fixed in 10% formaldehyde and embedded in paraffin according to standard procedures. The paraffin‐embedded samples were sectioned at 5 µm thickness using a rotary microtome (RM2255, Leica) and then stained with hematoxylin (MHS1, Sigma) and eosin (318906, Sigma) (H&E). The histology of lung and heart tissues was determined by light microscopy (Pannoramic MIDI and Pannoramic DESK, 3DHISTECH) and captured by the CaseViewer program (version 2.1, 3DHISTECH).

*Statistical Analysis*: The statistical differences between mean values obtained from the two sample groups were analyzed using Student's *t*‐test. The differences were considered significant if the *p* value was less than or equal to 0.05. The statistical differences between several samples types were analyzed with ANOVA, followed by the Newman–Keuls multiple comparison test. Asterisks (*, **, and ***) indicate the significance of *p* values (<0.05, <0.01, and <0.001, respectively).

## Conflict of Interest

The authors declare no conflict of interest.

## Supporting information

Supplementary

Supplementary

Supplementary

Supplementary
