# Supplementary material for: Mutual Destruction of Deep Lung Tumor Tissues by Nanodrug‐Conjugated Stealth Mesenchymal Stem Cells
Source: Adv Sci (Weinh). 2018 Feb 26;5(5):1700860. doi: 10.1002/advs.201700860 (PMC5979625; doi:10.1002/advs.201700860)
Supplement: Supplementary file 1 — Supplementary [file ADVS-5-1700860-s002.pdf]

Copyright WILEY-VCH Verlag GmbH & Co. KGaA, 69469 Weinheim, Germany, 2018.

Supporting Information

for *Adv. Sci.,* DOI: 10.1002/advs.201700860

Mutual Destruction of Deep Lung Tumor Tissues by Nanodrug-Conjugated Stealth Mesenchymal Stem Cells

Sang-Woo Kim, Yeon Kyung Lee, Jeong Hee Hong, Jun-Young Park, Young-Ae Choi, Dong Un Lee, Jungil Choi, Sun Jin Sym, Sang-Hyun Kim, and Dongwoo Khang**

Supplementary information for

Mutual Destruction of Malignant Lung Cancer by Nanodrug-Conjugated Stealth Mesenchymal Stem Cells

Sang-Woo Kim[1,*], Yeon Kyung Lee[1,*], Jeong Hee Hong[1,2,*], Jun-Young Park[1,*], Young-Ae Choi[4], Dong Un Lee[1], Jungil Choi[3], Sun Jin Sym[4], Sang-Hyun Kim[5,†], and Dongwoo Khang[1,2,†]

[1]*Lee Gil Ya Cancer and Diabetes Institute, Gachon University, Incheon 21999, South Korea*

[2]*Department of Physiology, School of Medicine, Gachon University, Incheon 21999, South Korea*

[3]*Gyeongnam Department of Environmental Toxicology and Chemistry, Korea Institute of Toxicology, Jinju 52834, South Korea*

[4]*Division of Hematology and Oncology, School of Medicine, Gachon University & Gil Hospital, Incheon 21565, South Korea*

[5]*Department of Pharmacology, School of Medicine, Kyungpook National University, Daegu 41566, South Korea*

[†]emails: dkhang@gachon.ac.kr or shkim72@knu.ac.kr

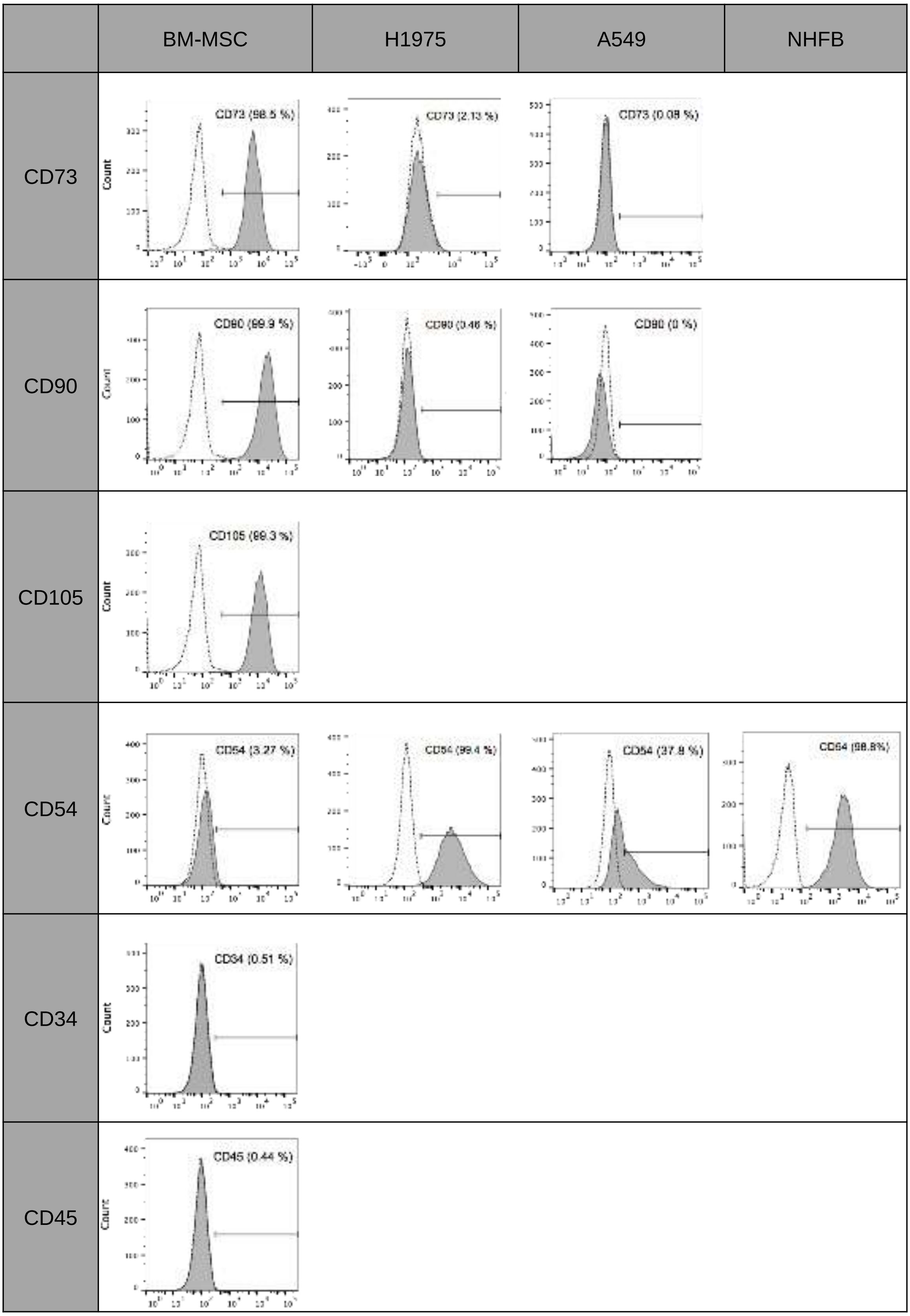

Supplementary Fig. 1. FACS analysis for identifying CD markers in BM-MSC, H1975**,** A549, and NHFB cells.

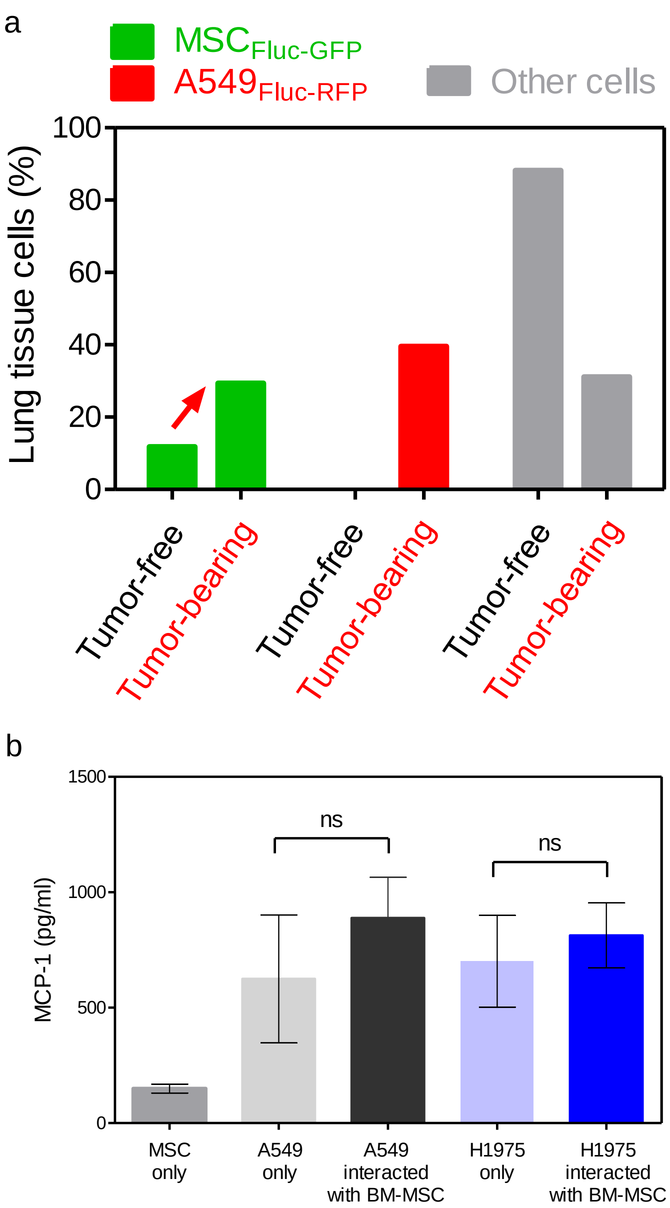

Supplementary Fig. 2. (a) Percentage of $MSC_{Fluc\text{-}GFP}$ that migrated to tumor-free and lung tumor-bearing ($A549_{Fluc\text{-}RFP}$) mice 2 days after the intravenous injection of $MSC_{Fluc\text{-}GFP}$. FACS analysis was performed to quantify the percentage of the lung tissue cells shown (from a total cell population of 10^4 cells). **(b)** MCP-1 chemokine from A549 and H1975 cells when co-cultured with BM-MSC. MCP-1 from lung cancer cells (i.e., A549 or H1975) did not increase when co-cultured with MSC. Data represent mean ± SEM (n=3) and the p- value reference was based on lung cancer (e.g., A549 and H1975) cells only (at 48 hrs).

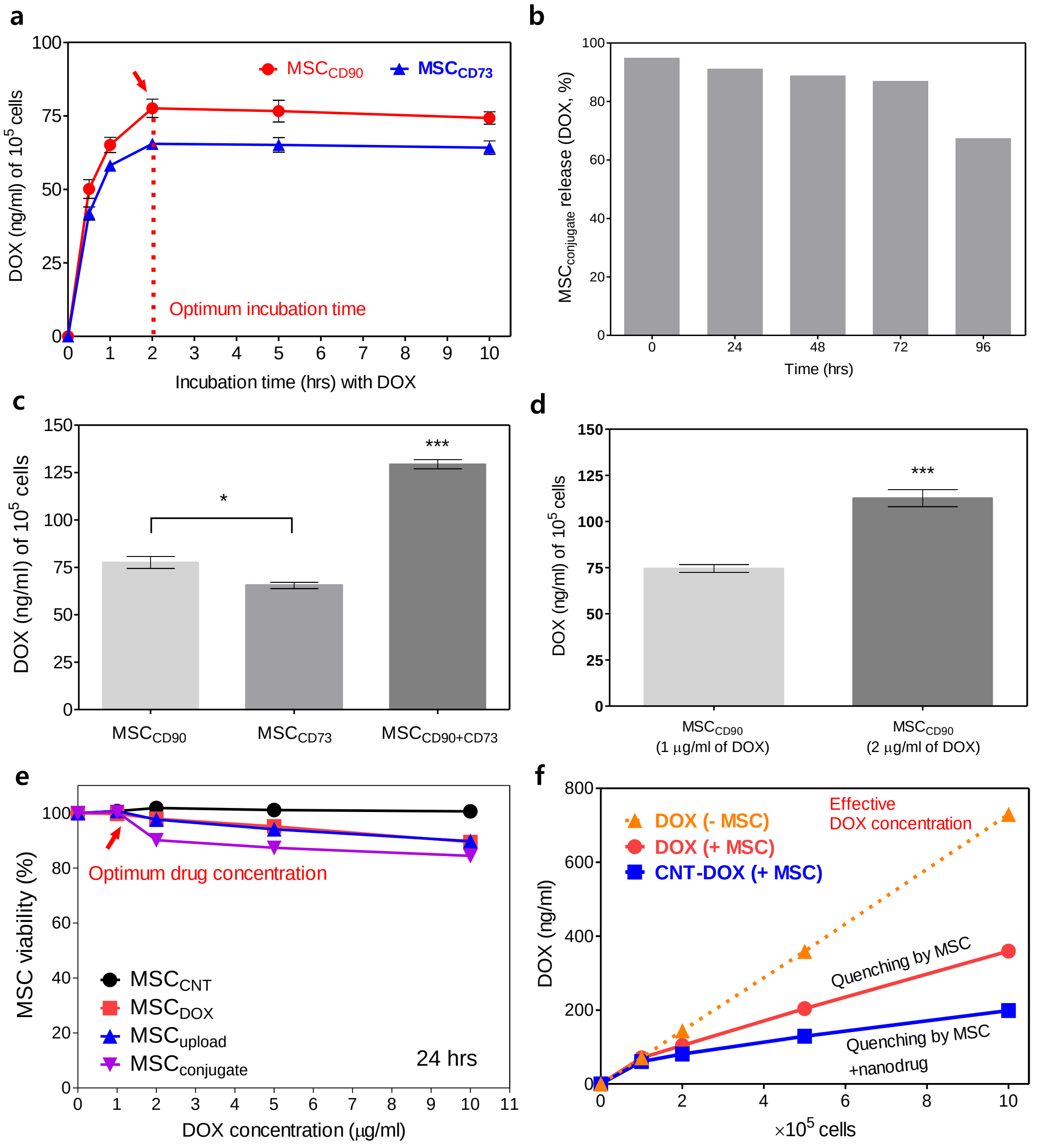

Supplementary Fig. 3. Optimization and stability of MSC-nanodrug conjugation. **(a)** Conjugated DOX concentration on MSC (10^5 cells) after various incubation time (i.e., 0-10 hrs) (at a dosage of 1 μg/ml of nanodrug treatment). **(b)** percentage of DOX release from $MSC_{conjugate}$ at up to 96 hrs (FACS). **(c)** DOX amount of MSC_{CD90}, MSC_{CD73}, and $MSC_{CD90+CD73}$ (at a dosing of 1 μg/ml of nanodrug treatment). **(d)** Amount of DOX conjugated to MSC_{CD90} after interaction with different drug concentrations (i.e., 1 or 2 μg/ml of nanodrug). **(e)** MTT assay confirmed that 1 μg/ml

of DOX is non-toxic for both MSC_{upload} and $MSC_{conjugate}$ after 24 hrs of incubation. Data represents mean ± SEM (n=5). **(f)** Graph represents the non-linear fluorescence standard curve of DOX. DOX fluorescence intensity (when mixed with MSC) was quenched by MSC and MSC-conjugated nanodrug. The excitation and emission wavelengths for DOX were 490 nm and 580 nm, respectively.

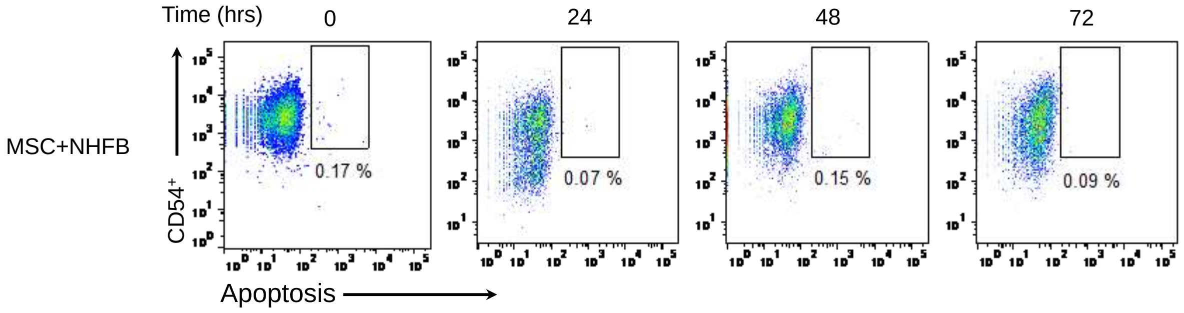

Supplementary Fig. 4. Time-dependent apoptosis of NHFB (normal cells) when co-cultured with MSC. Apoptosis of NHFB was found to be non-significant after 0, 24, 48 and 72 hrs (determined by BV421-labeled Annexin V, 1×10^4 single cell events were acquired).

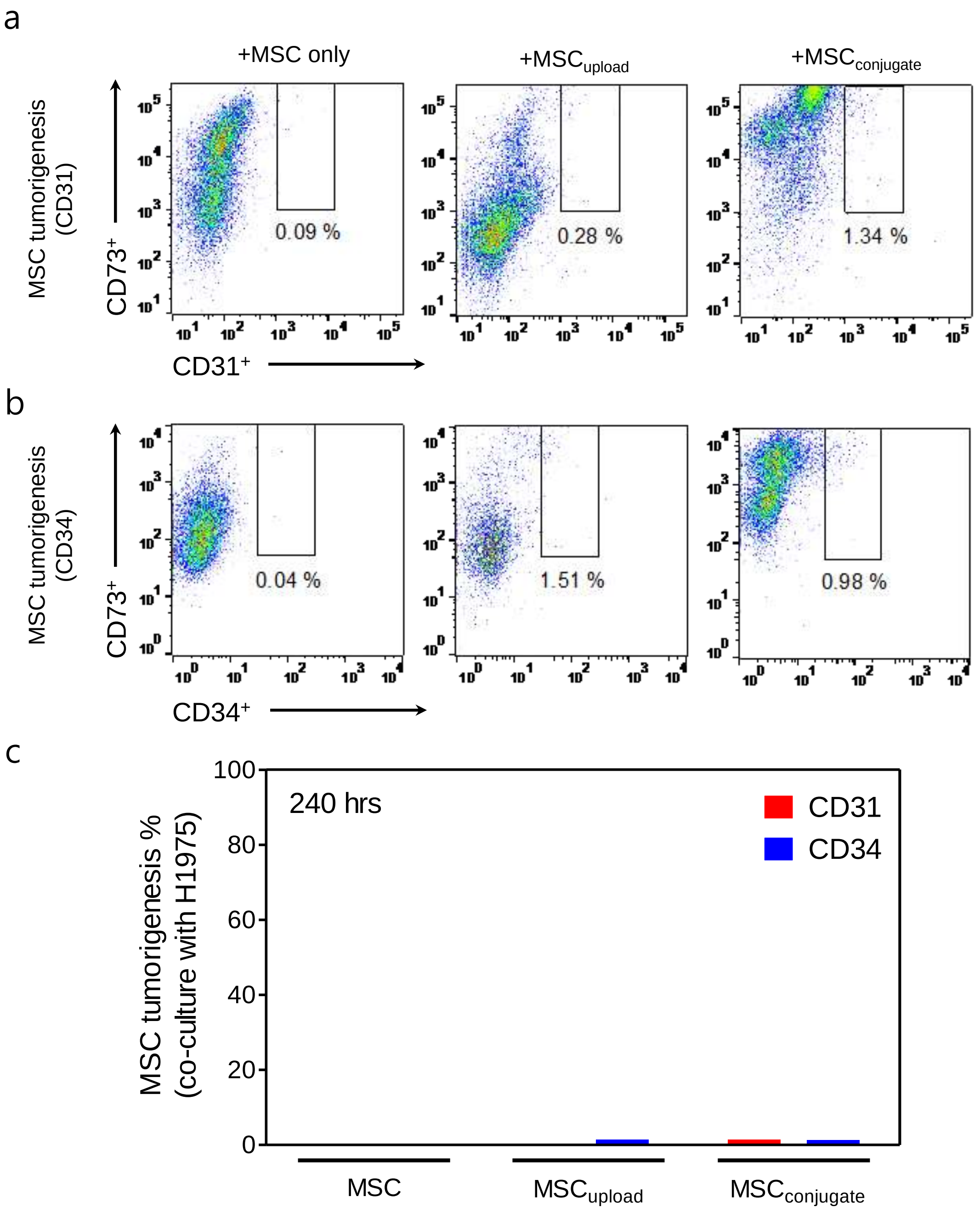

Supplementary Fig. 5. Long-term tumorigenesis of MSC, MSC_{upload} and $MSC_{conjugate}$ when co-cultured with H1975 lung cancer cells. After 240 hrs, tumorigenesis of MSC cells was identified by both BV421-labeled **(a)** CD31 and **(b)** CD34. (1×10^4 single cell events were acquired). **(c)** MSC tumorigenesis after 240 hrs of co-culture with H1975 cells confirmed negligible tumorigenesis markers (CD31 and CD34).

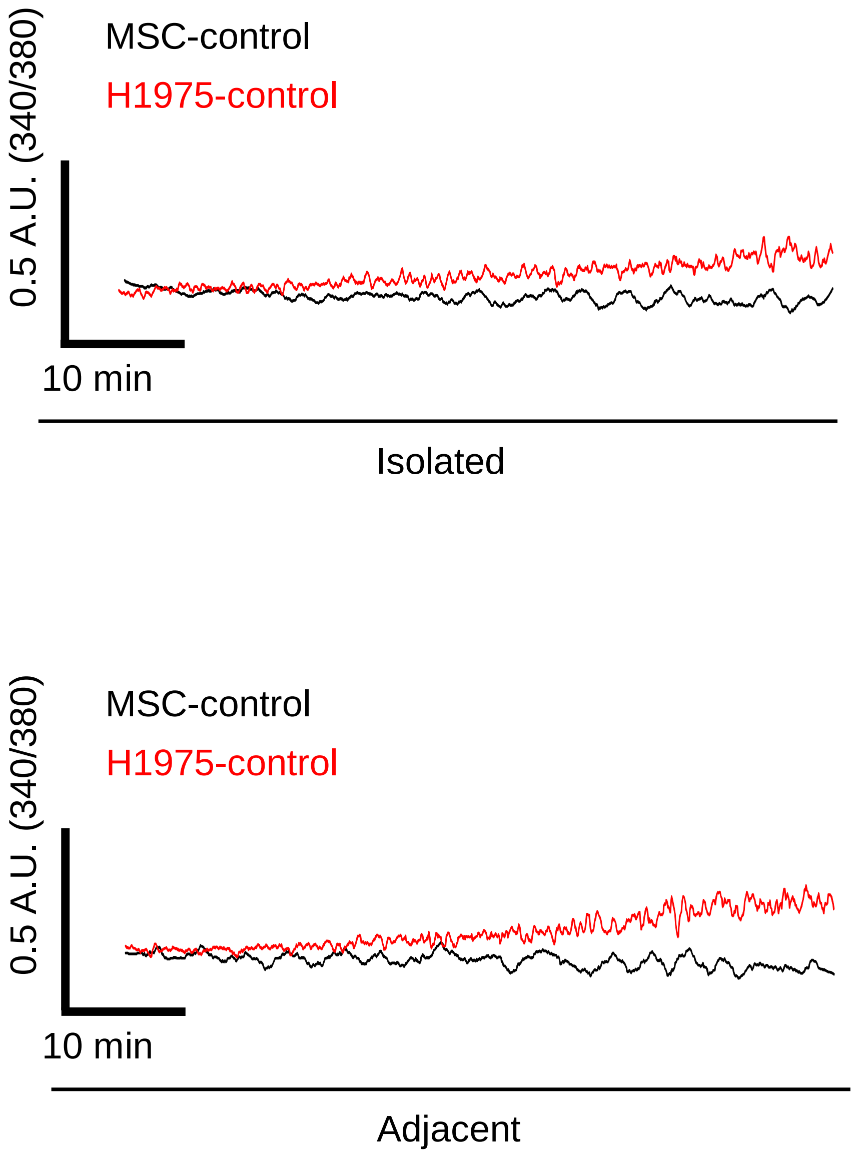

Supplementary Fig. 6. Changes in $[Ca^{2+}]_i$ for isolated and adjacent MSC-control (black) and H1975-control (red) cells.

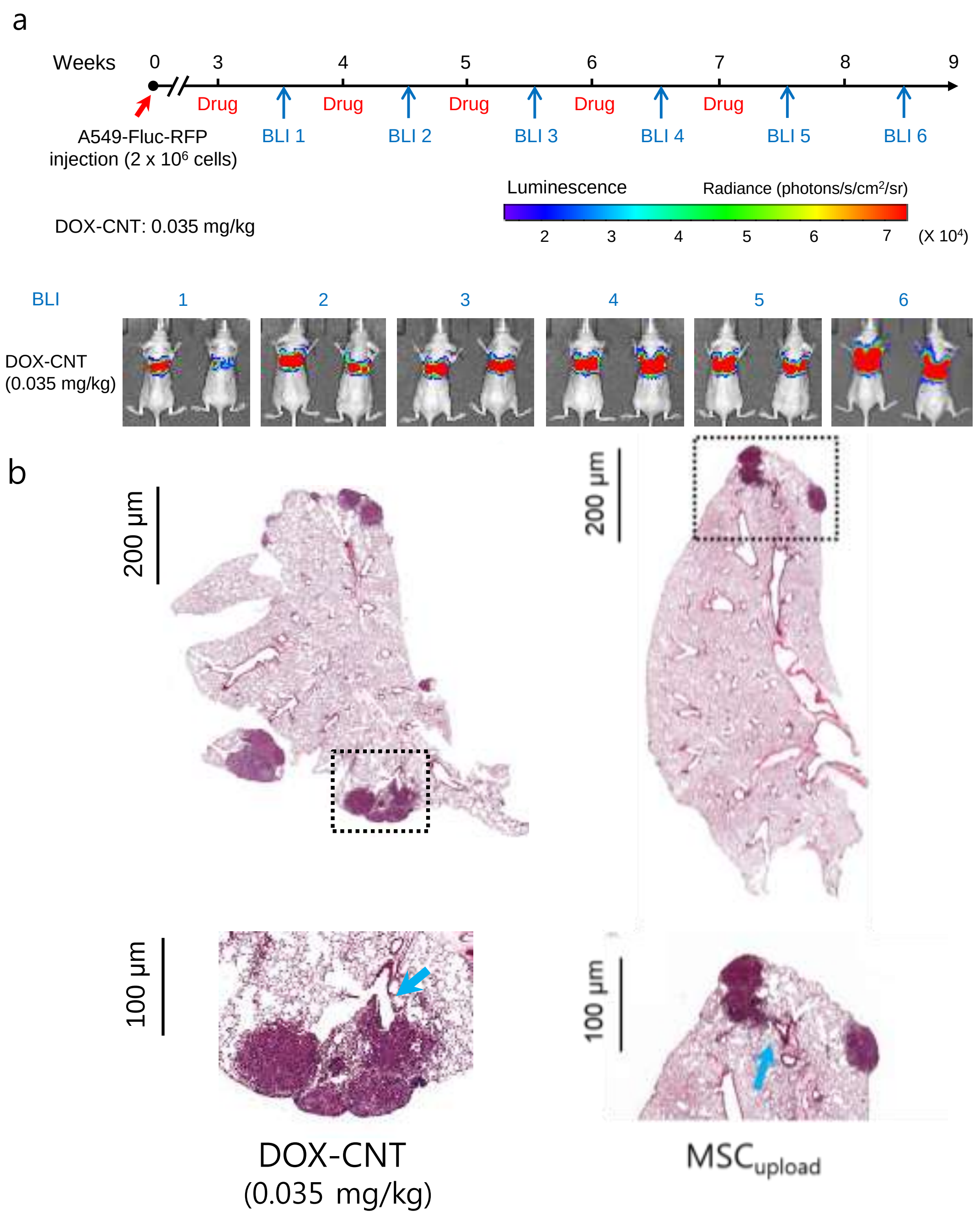

Supplementary Fig. 7. (a) BLI of luciferase expression of A549 lung tumor-bearing mice after treatment with DOX-CNT (0.035 mg/kg). **(b)** H&E staining (at 100x magnification) of lung tissue from A549 tumor-bearing mice after treatment with DOX-CNT (0.035 mg/kg) and MSC_{upload}. The dashed-box image shows the presence of tumor tissues; magnified view shows evidence of blood vessels connected to the tumor sites (indicated by a blue arrow).

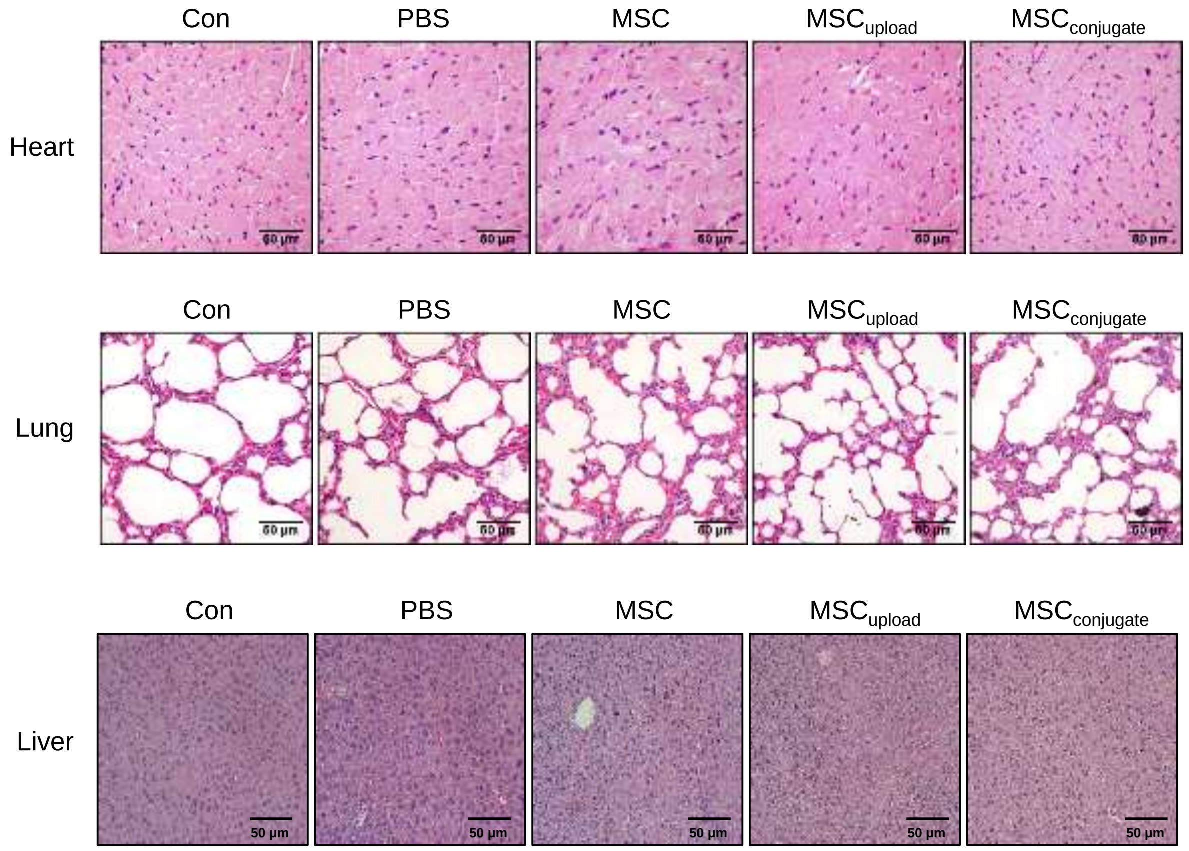

Supplementary Fig. 8. H&E-stained images of heart, lung, and liver tissues collected from mice with various injected treatments (i.e., control (con), PBS, MSC, MSC_{upload}, and $MSC_{conjugate}$) after 9 weeks (at 100x magnification).

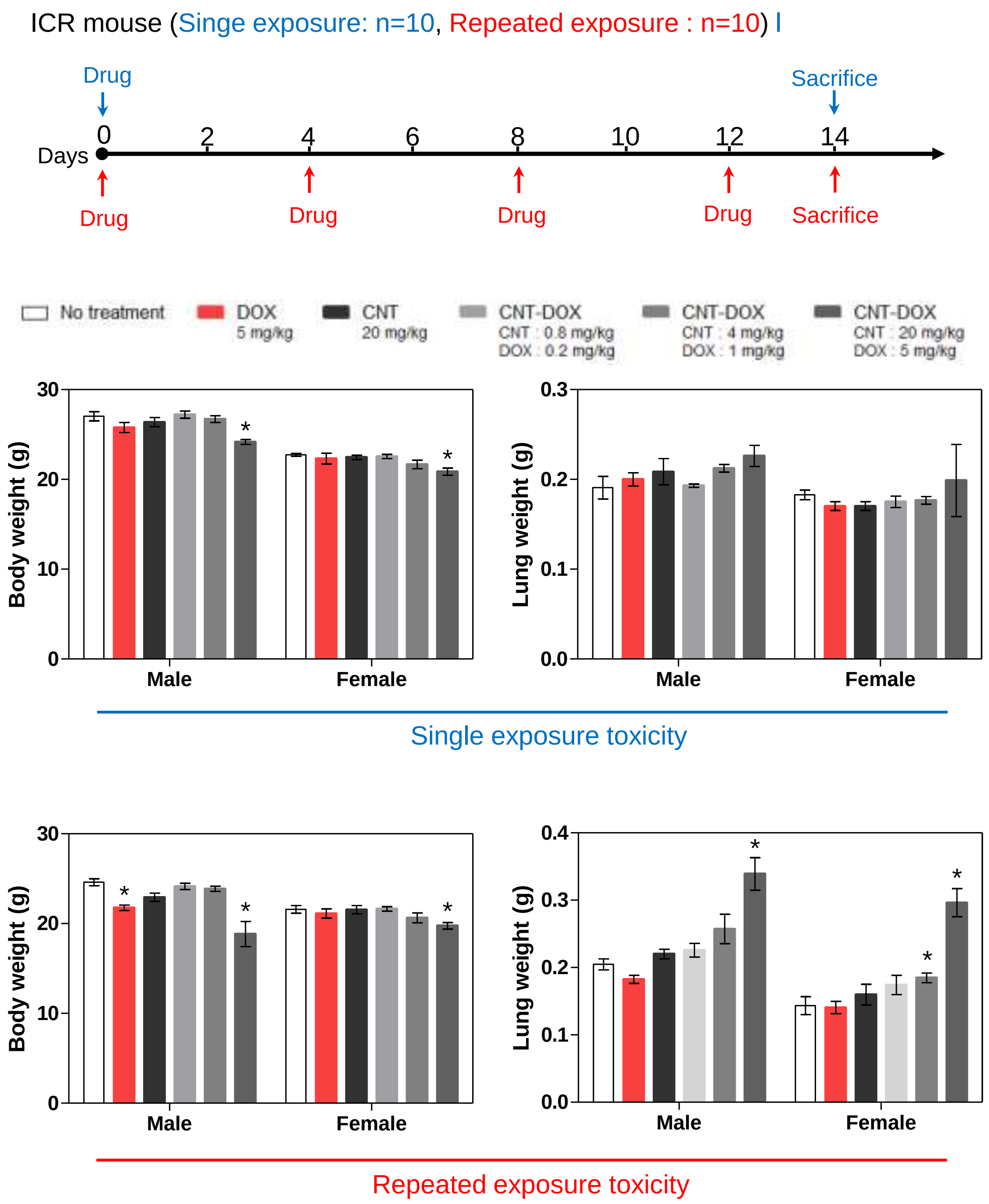

Supplementary Fig. 9. Single and repeated exposure toxicity evaluations shows significant lung weight changes in 5 mg/kg of CNT-DOX conjugation (20 mg/kg of CNTs with 5 mg/kg DOX conjugation). No pulmonary toxicity (weight change) was induced by nanodrug with dose of 0.2 mg/kg and 1 mg/kg of CNT-DOX (based on DOX dose).

Supplementary Movie 1: Live cell recording obtained by confocal quantitative image cytometer showing the mutual apoptosis of H1975 (green) cells and $MSC_{conjugate}$ (red) within 3~10 hrs of co-culturing.

Supplementary Movie 2: Live cell recording obtained by confocal quantitative image cytometer showing the mutual apoptosis of H1975 (green) cells near $MSC_{conjugate}$ (red) within 3~10 hrs of co-culturing.

Supplementary Movie 3: Live cell recording obtained by confocal quantitative image cytometer showing the no apoptosis of H1975 (green) cells near MSC (not seen) for 36 hrs of co-culturing.
